# A systematic review of reports on aquatic envenomation: are there global hot spots and vulnerable populations?

**DOI:** 10.1590/1678-9199-JVATITD-2024-0032

**Published:** 2024-12-20

**Authors:** Raechel Kadler, Catherine Pirkle, Angel Yanagihara

**Affiliations:** 1 Department of Tropical Medicine, Medical Microbiology and Pharmacology, John A. Burns School of Medicine, University of Hawai‘i at Mānoa, Honolulu, Hawaii, United States.; 2 Office of Public Health Studies, University of Hawai‘i at Mānoa, Honolulu, Hawaii, United States.; 3 Pacific Biosciences Research Center (PBRC), School of Ocean and Earth Science and Technology, University of Hawai‘i at Mānoa, Honolulu, Hawaii, United States.

**Keywords:** Envenomation, Aquatic, Toxin, Venom, Mortality, Morbidity, Epidemiology, Jellyfish, Catfish, Stingray

## Abstract

Envenomation by aquatic species is an under-investigated source of human morbidity and mortality. Increasing population density along marine and freshwater coastlines increases these incidents. Specific occupational groups - including commercial fishery workers, fisherfolk, marine tourism workers, and researchers - rely on aquatic resources for their livelihood. While diverse venomous aquatic species exhibit a broad array of habitats worldwide, they are most abundant in the tropics. Specific tropical regions present historic “hot spot” areas of concern for occupational groups with heightened risk of aquatic envenomation. Towards the overall objective of characterizing the health burden of aquatic envenomations, this review seeks to define (1) vulnerable, high-risk populations and (2) geographic hot-spot regions. To formally assess these metrics, a systematic literature review was performed where inclusion criteria requirements were peer-reviewed, published, epidemiological studies with defined denominators from January 1, 2000, to July 31, 2024, on the topic of human envenomation by aquatic species. Fifty-three articles met the inclusion criteria. Excluded articles were comprised of case reports, news and magazine articles, and those in languages aside from English, French, Portuguese, and Spanish. Most of the included articles examined emergency department and poison-control datasets that reported few overall envenomations (< 1%) from populations with physical and financial access to medical care. In contrast, datasets surveying beachgoers or fisherfolk directly, and life-guard incident reports, demonstrated that aquatic envenomation is an important source of injury for these groups and settings (envenomation frequency mean: 71%, median: 80%). Reports on additional high-risk groups, including marine and aquatic biologists, military personnel etc., and in key high-risk geographic regions including Thailand, Indonesia, and other Indo-Pacific countries were missing from the reviewed literature. Socio-demographic data were also largely missing from the literature. This systematic review highlights critical gaps where further research is needed, especially in under-represented regions and vulnerable populations.

## Background

Envenomation describes the delivery of a complex mixture of bioactive compounds, “venom”, by a bite or sting, which can lead to severe morbidity and mortality in human victims. There are venomous animals distributed globally including marine-dwelling animals such as jellyfish (Portuguese man-of-war/bluebottles, box jellyfish, “mauve stingers”, etc.), stingrays, lionfish, and freshwater animals such as certain species of catfish. Many of these can pose marked threats to humans. For example, since 2000, it is reported that box jellyfish have caused at least 14 deaths in Australia [1-7], 11 to 20 deaths in Thailand [8-16] and at least 10 deaths in the Philippines and Malaysia [17-24]. It is critical to point out that these citations represent documented case studies, but do not allow for the calculation of basic measures of burden of injury such as frequency, incidence, and prevalence due to a lack of denominator data (i.e., the population at risk).

The Global Burden of Disease, Injuries, and Risk Factors Study (GBD) describes the incidence, prevalence and human mortality due to diseases and injuries [25]. The causes are broadly categorized into three groups: communicable disease, non-communicable diseases, and injuries. Envenomation falls under injury, specifically in the unintentional injury category [25]. According to the 2019 GBD report, “venomous animal contact” caused 79,700 deaths in 2019, the vast majority of which were recorded in South Asia [25]. There was an approximately 4% increase in the prevalence and incidence rates of venomous animal contact between 2010-2019 [26].

There are limitations to the GBD studies on injury. The values reported in the GBD, and global surveillance of injury in general, are limited by the availability and quality of records. The majority of low-income countries use regional estimates and modeling data, categorized as the lowest quality level of data by the World Health Organization (WHO) [27]. This can result in dramatic inaccuracies, frequently in countries or regions, and within specific demographic groups, where envenomation is more likely to occur [27]. In a study of Canadian adults, unintentional injury was more likely among those with low education and income as compared to higher socioeconomic status (SES) [28]. Similarly, a systematic review demonstrated associations between unintentional injury and socioeconomic status among children and adolescents, highlighting the necessity for including health equity measures [29]. High-risk groups for snakebite include rural agricultural workers, hunters, and working children, as well as those with limited access to education and healthcare [30-35], exemplifying the need to identify vulnerable populations when conducting research on envenomation. Depending on the infrastructure for monitoring, as well as presence and quality level of reports, these pertinent health equity measures may not be captured. Further, deaths that occur without definitive or known causes can be categorized as “other unintentional injury” rather than “venomous animal contact”, contributing to the underestimation of cases [25]. Thus, accurate rates of global envenomation are lacking and the contribution of envenomation to the global burden of disease in many localities (e.g. the Philippines [36]) remains unknown.

Envenomation can be divided into two broad categories, terrestrial and aquatic. There is a critical gap in aquatic envenomation data compared to terrestrial, primarily snakebite, envenomations [30-33, 37]. Unlike snakebite, which was formally pronounced a neglected tropical disease (NTD) in 2017 and is now the subject of significant global surveillance and data collection, as well as working groups strategizing to reduce mortality and disability [34, 38], there is no similar mechanism for case incident reporting and record keeping for aquatic envenomations. Consequently, the current morbidity and mortality statistics are insufficient, largely due to a lack of resources and prioritization. 

Again, without infrastructure for incident reporting, such as mandated reporting in severe envenomation cases that require hospitalization or efforts by volunteer groups, many cases of aquatic envenomations go unrecorded or are inaccessible to the public [36, 39, 40, 41]. Envenomation events data may be recorded at various levels in the response and reporting chain. These differences (e.g. lifeguard notes, official patient record) and the importance ascribed to these events can affect how the incidents are categorized, and the level of description provided about them, as well as if they are formally memorialized in official records [40, 41]. This is exemplified by Brazil’s Information System for Notifiable Diseases (SINAN), where envenoming/injuries are recorded, but the specific description “type of animal - fish” was removed in 2007 and replaced by “accident type - other” [40]. Manual investigation is thus required to ascertain more details about the envenomation event, including the species [40]. This situation can be described as a “measurement trap” where the lack of data on health outcomes, its characteristics, and those affected by it, leads to its continued neglect [42].

It is important to identify the risk for aquatic envenomation, as 40% of the world's high-density populations reside within 100 km of coastal zones where marine resource related occupations are highly prevalent [43]. Interactions with venomous aquatic organisms are likely higher for Indigenous Peoples [44, 45, 46]. Per capita global seafood consumption for Indigenous Peoples is 15 times higher than non-Indigenous populations [45]. Further, their consumption is highest in tropical regions, where venomous fish fauna is most abundant, therefore posing an increased risk of exposure for those who harvest them [46]. Fisherfolk, populations that bathe in rivers or oceans, children that play in shallow waters, and those in the marine-tourism industry are at increased risk of exposure [36, 41, 47]. Yet, quantitative data on geographic hot-spots and high-risk populations of aquatic envenomation are lacking. 

The objective of this systematic review is to characterize the health burden of aquatic envenomations. It seeks to (1) identify high-risk geographical regions and (2) the characteristics of populations more likely to experience envenomation. To ensure a focus on health equity among envenomation victims and prevent neglect of affected populations, equity reporting guidelines were followed, and demographics were included as applicable [48, 49]. We aim to provide numbers on the amplitude of the problem; identify where and for whom programs are needed to prevent and manage envenomation injury and assess if there is a need for expanded research on the topic.

## Methods

### Search strategy and analysis

*Search terms*

This systematic review was performed to identify articles on the topic of aquatic envenomation. The search terms selected to capture the action of envenomation include: “Stings”, “Bites”, “Envenomation”, “Toxin”, “Poison”, or “Venom.” To capture measures of the burden of envenomation we searched the following epidemiological terms: “Mortality”, “Morbidity”, “Burden”, “Prevalence”, “Epidemiology”, or “Incidence.” Finally, search terms included a list of known venomous animals. The full search terms can be found in the Additional file 1. All terms were in English. We used the PubMed, AGRICOLA, SciELO, and Web of Science databases to collect reports published between January 1, 2000, and July 31, 2024. We consulted with a public health librarian to select and finalize search terms and databases.

*Strategy*

There were a total of 4349 results from PubMed, 2226 from Web of Science, 173 from SciELO, and 663 from AGRICOLA, summing 7411 total articles. Records from each database were imported into Zotero, a reference management system, and deduplicated. Then, the files were imported into Rayyan, and any additional duplicates were detected and removed. A total of 6237 unique articles remained.

*Inclusion and exclusion criteria*

Inclusion criteria and exclusion criteria are presented in Table 1. While search terms were in English, articles in English, French, Spanish and Portuguese were included, while all other languages were excluded. Many non-English language articles are indexed in the searched databases. The specific envenoming animal was limited to aquatic species and included stings and bites, but not ingestion or inhalation.

**Table 1.** Inclusion and exclusion criteria.

Criteria	Include	Exclude
Publication date	January 1, 2000, to July 31, 2024	Prior to 2000 or after July 2024
Study design	Observational epidemiological studies: Prospective/retrospective cohort studies, case-control studies, cross-sectional studies (e.g., surveys, analysis of surveillance data)	Case reports, anecdotal reports
Type of publication	Peer-reviewed journals	Textbooks, reports, newspapers, magazines, encyclopedias
Language	English, French, Spanish, Portuguese	All other languages
Population (envenomed)	Human	Animal
Animals (envenomation)	Aquatic venomous species	Terrestrial venomous species, non-venomous species, poisonous species
Envenomation	Sting, bite	Ingestion, inhalation

*Article screening*

Following deduplication, the title and abstracts of the remaining articles were reviewed independently by a primary reviewer (RK) with a secondary reviewer (CP) confirming inclusion criteria were met. There were 6237 articles after deduplication, of which 1153 were specific to aquatic envenomation and further screened. Full texts (159) were screened by two reviewers (RK and CP) independently, while potential undetermined reports were analyzed by both reviewers until a consensus was reached. While screening the articles, backwards and forwards citation searching was used to identify additional reports that were not identified in the original search outputs.

*Data extraction*

Data extracted from the reports included study characteristics such as the country of study, geographical setting, study objective and design, sampling strategy, data collection method, as well which envenomating species, genus, and/or family was discussed. This was followed by extraction of quantitative indicators of envenomation by animals or groups of animals, such as prevalence estimates and/or incidence. When available, the prevalence and incidence was categorized further by demographic characteristics of the victims including age, sex, race, occupation, religion, education, and SES, in accordance with PROGRESS-Plus [48, 49]. PROGRESS-Plus is an acronym utilized to identify characteristics about the population being observed to determine contributing factors to the outcome. It provides a framework to organize information and delineate associations [49]. The management, outcome, and consequences (mortality and morbidity) of envenomation from each species were also included when possible.

## Results

**Figure 1.**
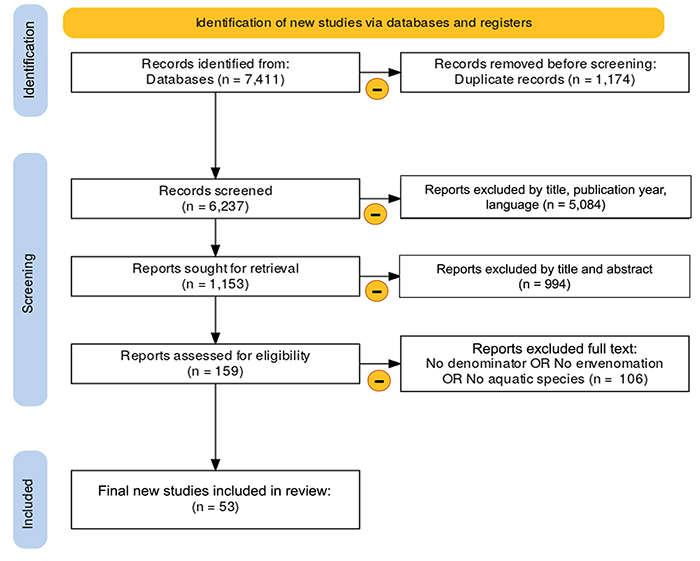
PRISMA flow diagram shows inclusion and exclusion criteria.

A total of 53 articles were included in this review. Figure 1 provides a flow-chart of the numbers of articles identified, screened and included in this review. Table 2 provides details about the papers, including the geographic setting, study objective and design, sampling procedures, and the envenomating organisms investigated, including the numbers and/or percentages of envenomating events recorded in the study [36, 40, 41, 47, 50-98].

**Table 2.** Characteristics of studies included in the present work.

Title	Author(s), year published, ref.	Geographic setting	Years of data	Study objective	Study design	Sampling strategy & data collection method	Organisms	Bite or sting number & percent
North America								
Animal-related fatalities in the United States - an update	Langley RL, 2005 [50]	United States	1991-2001	Evaluate the causes of human fatalities due to animal encounters in the United States between 1991 to 2001	Cross-sectional	CDC Wonder database was used to query data from the center between 1991-2001. Fatalities between 1991 to 1998 coded by ICD-9 edition (E-905.6 = venomous marine animals and plants) and from 1999-2001 ICD-10 edition (X26 = venomous marine animals and plants)	Venomous marine animals Unspecified venomous animal Other	2 (0.1%) 23 (1.2%) 1 (0.05%)
Pattern of stingray injuries reported to Texas poison centers from 1998 to 2004	Forrester MB, 2005 [51]	Texas, United States	1998-2004	Examine the relationship between selected factors and all human exposures involving stingray injuries reported to Texas poison centers	Cross-sectional	The Texas Poison Center Network (TPCN) consists of six poison control centers, that collect information on all calls into the Toxic Exposure Surveillance System (TESS). Cases involving stingrays were investigated and the penetrance (cases per 1000 population) were calculated	Stingrays	Year, Cases, Penetrance 1998 - 12 (0.0006) 1999 - 17 (0.0008) 2000 - 15 (0.0007) 2001 - 27 (0.0013) 2002 - 30 (0.0014) 2003 - 28 (0.0013) 2004 - 24 (0.0011)
Skin problems related to the occupation of commercial fishing in North Carolina	Burke WA, et al., 2006 [52]	Eastern North Carolina, USA	2002-2004	Elucidate the types of occupational skin disorders that occur in commercial fishermen in North Carolina	Cross-sectional	A convenience sample was used. Booths were set up offering free skin cancer screening at various seafood festivals including a "blessing of the fleet" event and commercial fishing shows, located throughout eastern North Carolina. When screened, fishermen were asking about history of skin lesions.	Bites/stings Jellyfish Portuguese man-of-war Stingrays Fish stings (spiny dogfish and sea catfish)	69 (85%) 22 (27%) 9 (11%) 26 (32%) at least 1 15 (19%) >1 5 (6%)
Epidemiology of non-canine bite and sting injuries treated in U.S. emergency departments, 2001-2004.	O'Neil ME, et al., 2007 [53]	United States	2001-2004	Estimate the burden of non-canine related bite and sting injuries; described the affected population; injury severity; bite or sting source; and provide considerations for prevention strategies	Cross-sectional	Data extraction from the National Injury Surveillance System - All Injury Program. This is a nationally representative sample of 66 hospitals. The system tracks all injuries seen in emergency departments. They calculated the weighted annual national estimate for being treated in emergency departments.	Jellyfish Stingray Other marine animals	724 (0.1%) 2459 (0.3%) 829 (0.1%)
Animal bites and stings reported by United States poison control centers, 2001-2005	Langley RL, 2008 [54]	United States	2001-2005	Provide information on the frequency of occurrence of injuries from animals (in instances where individuals may not visit a health care center, but do contact poison control centers)	Cross-sectional	The American Association of Poison Control Centers (AAPCC) annual reports were reviewed and summarized on different species of animal bites and stings (92,829 total)	Coelenterates (jellyfish, sea anemone, corals) Fish Other/unknown Total	1046 (1.12%) 1295 (1.40%) 493 (0.53%) 2834 (3.05%)
2011 Annual Report of the American Association of Poison Control Centers' National Poison Data System (NPDS): 29th Annual Report	Bronstein AC et al., 2012 [55]	United States	2011	Summarize calls made to America's Poison Centers, documented in the National Poison Data System (NPDS)	Cross-sectional	Data extraction from NPDS	Aquatic ENV Fish stings Cnidaria Other/unknown	1768 (2.7% ENV, 0.08% TC) 888 (1.3% ENV, 0.04% TC) 539 (0.8% ENV, 0.02% TC) 341 (0.5% ENV, 0.01% TC)
Fatalities from Venomous and Nonvenomous Animals in the United States (1999-2007)	Forrester JA et al., 2012 [56]	United States	1999-2007	To review recent US mortality data from deaths caused by nonvenomous and venomous animals and compare recent data with historic data	Cross-sectional	The CDC Wonder database was used to query data from all animal-related fatalities between 1999-2007 (ICD-10 codes W53-W59 and X20-X29)	Venomous marine animals and plants	1 (0.1%)
The Toxicology Investigators Consortium Case Registry - The 2011 Experience	Wiegand TJ et al., 2012 [57]	United States	2010-2011	Toxico-surveillance and research to fulfill two gaps in the field: real-time toxico-surveillance system to identify current poisoning trends and a powerful research tool in toxicology	Prospective registry	Prospective registry of all cases seen by medical toxicologists at participating institutions and recorded into online database were analyzed. Fields were populated for each patient.	Portuguese man-of-war (jellyfish)	1 (0.34%)
The Toxicology Investigators Consortium Case Registry - The 2012 Experience	Wiegand TJ et al., 2013 [58]	United States	2010-2012	Toxico-surveillance and research to fulfill two gaps in the field: real-time toxico-surveillance system to identify current poisoning trends and a powerful research tool in toxicology	Prospective registry	Prospective registry of all cases seen by medical toxicologists at participating institutions and recorded into online database were analyzed. Fields were populated for each patient.	Portuguese man-of-war (jellyfish) *Pterois* sp. (lionfish)	1 (0.2% ENV, 0.0057% TC) 2 (0.41% ENV, 0.011% TC)
National Estimates of Noncanine Bite and Sting Injuries Treated in US Hospital Emergency Departments, 2001-2010	Langley RL et al., 2014 [59]	United States	2001-2010	Quantify nonfatal bite and sting injuries by non-canine sources using data from the National Electronic Injury Surveillance System- All Injury Program. It provides an update on the work of O’Neil et al.	Cross-sectional	Data extraction from the National Injury Surveillance System- All Injury Program. This is a nationally representative sample of 66 hospitals. The system tracks all injuries seen in emergency departments.	Jellyfish Stingray Fish Other marine animals	786 (0.1%) 2150 (0.2%) 627 (0.1%) 638 (0.1%)
2015 Annual Report of the American Association of Poison Control Center' National Poison Data System (NPDS): 33rd Annual Report	Mowry JB et al., 2016 [60]	United States	2015	Summarize calls made to America's Poison Centers, documented in the National Poison Data System (NPDS)	Cross-sectional	Cross-sectional	Aquatic ENV Fish stings Cnidaria Other/unknown	1220 (2.4% ENV, 0.06% TC) 619 (1.2% ENV, 0.03% TC) 327 (0.6% ENV, 0.02% TC) 274 (0.5% ENV, 0.01% TC)
Mortality, hospital admission, and healthcare cost due to injury from venomous and non-venomous animal encounters in the USA: 5-year analysis of the National Emergency Department Sample	Forrester JD et al., 2018 [61]	United States	2010-2014	Characterize animal-related injuries presenting to US emergency departments (ED) to determine the impact of these types of injuries	Cross-sectional	All ED encounters with diagnosis codes corresponding to animal related injuries were identified from the National Emergency Department Samples (NEDS)	Venomous marine animals and plants	34,871 (1%)
Animal-encounter fatalities: United States, 1999-2016: Cause of death and misreporting	Haskell MG and Langley RL, 2020 [62]	United States	1999-2016	Characterize and compare fatalities by animal-encounter mentions reported by underlying cause of death (UCD) and multiple cause of death (MCD) to determine factors associated with misreporting UCD	Cross-sectional retrospective descriptive analysis	Analyzed fatality data from CDC Online Data for Epidemiological Research	Venomous marine animals and plants	UCD = 1 (0.03%) MCD = 2 (0.05%)
National Estimates of Noncanine Bite and Sting Injuries Treated in US Hospital Emergency Departments, 2011-2015	Hareza D et al., 2020 [63]	United States	2011-2015	Quantify and update the nonfatal bite and sting injuries by noncanine sources with the purpose of using these updates to better understand public health consequences and prevention techniques	Cross-sectional	Data extraction from the National Injury Surveillance System - All Injury Program. This is a nationally representative subsample of NEISS hospitals	Marine total Jellyfish Stingray Catfish Lionfish Scorpion fish Sculpin Other marine animals	4275 (0.4%) 748 (17.5%) 2312 (54.1%) 220 (33.7%) 55 (8.4%) 27 (4.2%) 53 (8.1%) 562 (13.1%)
Envenomations during pregnancy reported to the national poison data system, 2009-2018	Ramirez-Cruz MP et al., 2020 [64]	United States	2009-2018	Characterize the clinical effects, treatments, and outcomes of envenomations during pregnancy reported to poison control centers across the US during a recent 10-year period	Cross-sectional retrospective descriptive study and case-control	Data extraction from NPDS. Case control of non-pregnant women of childbearing age (15-44 years)	Marine total Jellyfish/other coelenterates Fish stings Other unknown marine animals	Pregnant Non-pregnant 47 (1.3%) 2092 (2.4%) 27 (0.8%) 911 (1.0%) 19 (0.5%) 978 (1.1%) 1 (0.03%) 203 (0.2%)
2020 Annual Report of the National Poison Data System© (NPDS) from America's Poison Centers: 38th Annual Report	Gummin DD et al., 2021 [65]	United States	2020	Summarize calls made to America's Poison Centers, documented in the National Poison Data System (NPDS)	Cross-sectional	Data extraction from NPDS	Aquatic ENV Fish stings Cnidaria Other/unknown	1439 (3.4% ENV, 0.07% TC) 620 (1.47% ENV, 0.03% TC) 265 (0.6% ENV, 0.01% TC) 554 (1.31% ENV, 0.03% TC)
2021 Annual Report of the National Poison Data System© (NPDS) from America's Poison Centers: 39th Annual Report	Gummin DD et al., 2022 [66]	United States	2021	Summarize calls made to America's Poison Centers, documented in the National Poison Data System (NPDS)	Cross-sectional	All 55 USA PCCs documented received calls to the NPDS, where data was extracted from for this report. The 50 United States, American Samoa, the District of Columbia, Federated States of Micronesia, Guam, Marshall Islands, Northern Marianas, Puerto Rico, and the US Virgin Islands were included.	Aquatic ENV Fish stings Cnidaria Other/unknown	1248 (3.5% ENV, 0.06% TC) 488 (1.36% ENV, 0.02% TC) 259 (0.7% ENV, 0.012% TC) 501 (1.4% ENV, 0.024% TC)
Nationwide Aquatic Envenomations Reported to US Poison Control Centers from 2011 to 2020	Kirchberg TN et al., 2024 [67]	United States	2011-2020	Describe the aquatic envenomation exposures occurring in the United States between 2011 and 2020 to better understand the epidemiology and assess any trends over time	Cross-sectional	Data was extracted from the Association of Poison Control Centers (AAPCC) National Poison Data System (NPDS) for all aquatic envenomations reported during 2011 to 2020. The number of centers reporting varied from 55 to 57 during this time. Incidence of envenomation per state calculated using US Census data.	Aquatic ENV Fish stings Cnidaria Other/Unknown	8519 (0.04% TC) 5159 (61% AQENV, 0.02% TC) 2519 (30% AQENV, 0.01% TC) 839 (10% AQENV, 0.004% TC) Rate of envenomation = 2.8 per 100,000 residents
South America								
Puncture wounds by driftwood catfish during bucket baths: local habits of riverside people and fish natural history in the Amazon	Sazima I et al., 2005 [68]	Solimoes River, near Manaus, Central Amazon, Brazil	Does not report	To assess the prevalence of stings by small spiny driftwood catfish (“*carataí*”) of the genus *Centromochlus* (Auchenipteridae) accidentally caught in buckets during bucket bathing by riverside people along the Brazilian Amazon	Cross-sectional	Convenience sample of riverside dwellers were interviewed; about 10% of the population in that area.	Driftwood catfish (*Centromochlus existimatus* and *C. heckelii*, “*carataí*”)	17 (63%)
Fauna attacks in French Guiana: a retrospective 4-year analysis	Mimeau E and Chesneau P, 2006 [69]	French Guiana	1998-2001	To document and characterize the risks of fauna attacks in French Guiana. Describe which animals are most often involved and what human populations are at risk	Cross-sectional retrospective	Records from the Service d'Aide Médicale Urgente (SAMU) were examined. All calls made to the Centre de Reception et Regulation de Appels (CRRA) that involved animal encounters were included. The information recorded included victim characteristics, type of animal that caused the injury, and management decisions	Cnidarians Fish (may be venomous)	4 (0.6% ABS, 0.005% TC) 24 (3.6% ABS, 0.03% TC)
Injuries and envenomings by aquatic animals in fishermen of Coxim and Corumbá municipalities, State of Mato Grosso do Sul, Brazil: Identification of the causative agents, clinical aspects and first aid measures	Silva GC et al., 2010 [47]	Mato Grosso do Sul State: Coxim and Corumbá, Brazil	2008-2009	Study injuries in professional fishermen	Cross-sectional	An interview was completed alongside a questionnaire with a random sample of fishermen	Catfish + Stingrays	78 (78%)
							Catfish (*Pimelodus* sp., *Pimelodella* sp., *Rhamdia* sp.)	Coxim = 10 (10%) Corumbá = 13 (13%)
							*“Jurupensém”* or *“sorubim lima”*	Coxim = 9 (9%) Corumbá = 3 (3%)
							“*Jurupoca*” *(Hemisorubin platyrhynchos)*	Coxim = 3 (3%) Corumbá = 0 (0%)
							“*Sorubim*”, catfish (*Pseudoplatystoma* sp.)	Coxim = 14 (14%) Corumbá = 10 (10%)
							Stingrays (*Potamotrygon motoro,* *P. falkneria,* *P. brachyura*)	Coxim = 7 (7%) Corumbá = 9 (9%)
Overall pattern of accidents caused by poisonous animals in Colombia, 2006-2010	Rodríguez-Vargas AL et al., 2012 [70]	Colombia	2006-2010	Establish a baseline concerning accidents by poisonous animals reported via telephone to the Toxicology Management and Research Information Centre (CIGITOX) to promote public health programs and raise awareness	Cross-sectional retrospective descriptive study	Information from telephone calls concerning accidents that were recorded in the CIGITOX database were extracted and analyzed	Aquatic animals (anemones, rays, sea urchins, and jellyfish)	26 (0.07% TC, 1.5% ENV)
Trauma and envenoming caused by stingrays and other fish in a fishing community in Pontal do Paranapanema, State of São Paulo, Brazil: epidemiology, clinical aspects, and therapeutic and preventive measures	Haddad Junior V et al., 2012 [71]	Rosana communit, Paraná River, Brazil	Does not report	Establish a clinical and epidemiological profile of accidents with stingrays and other fish	Cross-sectional	A questionnaire was completed by a convenience sample	Stingrays “*Mandijubas*” (*Pimelodus maculatus*) *“Surubins”* (spotted catfish)	6 (15.4%) 25 people, > 84 incidents (> 53.8%) 9 (1 person stung > 3 times) (> 30.8%)
Mortality caused by venomous animals in Venezuela: 1980-1999	De Sousa L et al., 2014 [72]	Venezuela	1980-1999	Describe the epidemiological pattern of human mortal accidents caused by venomous animals in Venezuela	Cross-sectional	National mortality data was extracted from the Venezuelan National Health System through inspection of series E905 and X20 to X29. “Other animals” included codes E905.6 to E905.9, and X26 to X29. Venomous marine animals and plants = X26.	Other animals	58 (3.9%) **Year - Frequency - Incidence (/100,000)** 1980 - 1 (1.7%) - 0.01 1981 - 6 (10.3%) - 0.04 1982 - 3 (5.2%) - 0.02 1983 - 1 (1.7%) - 0.01 1984 - 0 - 0 1985 - 1 (1.7%) - 0.01 1986 - 3 (5.2%) - 0.02 1987 - 0 - 0 1988 - 2 (3.4%) - 0.01 1989 - 4 (6.9%) - 0.02 1990 - 4 (6.9%) - 0.02 1991 - 4 (6.9%) - 0.02 1992 - 0 - 0 1993 - 1 (1.7%) - 0.005 1994 - 3 (5.2%) - 0.01 1995 - 0 - 0 1996 - 10 (17.2%) - 0.04 1997 - 7 (12.1%) - 0.03 1998 - 4 (6.9%) - 0.02 1999 - 4 (6.9%) - 0.02
Injuries caused by aquatic animals in Brazil: an analysis of the data present in the information system for notifiable diseases	Reckziegel GC et al., 2015 [40]	N, NE, SE, S, and Central West Brazil	2007-2013	Review of injuries in humans caused by aquatic animals in Brazil, using the information system for notifiable disease (SINAN)	Cross-sectional	All available data from SINAN records were used. SINAN records filtered from accident type - other and divided into six groups of aquatic animals contained in the SINAN: jellyfish/Portuguese man-of-war (PMW), stingray, catfish, toadfish, sea urchin, other Other includes accident by fish, fish sting, marine fish, “*ictismo*” (Brazilian term for accident by fish), *“tilápia’, “piranha”, “traíra”*, moray eel	Venomous aquatic animals	3651 (88.7%)
							Jellyfish/PMW	2007 - 114 (25.3%) 2008 - 127 (22.4%) 2009 - 75 (13.1%) 2010 - 26 (4.9%) 2011 - 43 (7.8%) 2012 - 47 (6.2%) 2013 - 108 (15.6%) total = 540 (13.1%)
							Stingrays	2007 - 193 (42.9%) 2008 - 331 (58.5%) 2009 - 387 (67.5%) 2010 - 405 (76.6%) 2011 - 429 (77.7%) 2012 - 624 (82.5%) 2013 - 473 (68.4%) total = 2842 (69%)
							Freshwater stingrays	2603 (63%)
							Toadfish	2007 - 18 (4%) 2008 - 14 (2.5%) 2009 - 26 (4.5%) 2010 - 33 (6.2%) 2011 - 29 (5.3%) 2012 - 25 (3.3%) 2013 - 36 (5.2%) total = 181 (4.4%)
							Catfish	2007 - 9 (2%) 2008 - 6 (1.1%) 2009 - 6 (1%) 2010 - 9 (1.7%) 2011 - 4 (0.7%) 2012 - 14 (1.9%) 2013 - 23 (3.3%) total = 71 (1.7%)
							Sea urchins	2007 - 3 (0.7%) 2008 - 2 (0.4%) 2009 - 3 (0.5%) 2010 - 2 (0.4%) 2011 - 2 (0.4%) 2012 - 1 (0.1%) 2013 - 4 (0.6%) total = 17 (0.4%)
							Other	2007 - 113 (25.1%) 2008 - 86 (15.2%) 2009 - 76 (13.3%) 2010 - 54 (10.2%) 2011 - 45 (8.2%) 2012 - 45 (6%) 2013 - 48 (6.9%) total = 467 (11.3%)
Injuries caused by the venomous catfish pintado and cachara (*Pseudopltaystoma* genus) in fishermen of the Pantanal region in Brazil	Aquino GN et al., 2016 [73]	Mato Grosso du Sul State: Miranda and Corumbá, Brazil	2013	Occurrence of injuries caused by fish of the *Pseudoplatystoma* genus, identifying causes, predisposing factors, aspects of the injuries, and physical and socioeconomic consequences	Cross-sectional	Not stated, presumably convenience sample. Interviews with a questionnaire were completed	Total *Pseudoplatystoma corruscans* (spotted catfish "*pintado*") Pseudoplatystoma reticulatum (striped catfish "*cachara*")	149 (30.98%) 76 (51% ENV, 15.8% TC) 73 (49% ENV, 15.2% total)
Injuries caused by freshwater stingrays in the Tapajos River Basin: clinical and sociodemographic study	Abati PAM et al., 2017 [74]	Riverine communities and the Extractive reserve if Tapajos-Arapiuns, Brazil	2010	Identify the sociodemographic, clinical and therapeutic aspects related to stingray injuries	Cross-sectional	Interviews with a questionnaire were completed from a convenience sample	Freshwater stingrays (*Potamotrygonid* group)	19 (6.3%)
Delayed healthcare and secondary infections following freshwater stingray injuries: risk factors for a poorly understood health issue in the Amazon	Sachett J et al., 2018 [75]	State of Amazonas: Alvares, Uarini, and Silves, Brazil	2007-2014	Aims to describe the profile of freshwater stingray injuries and risk factors for secondary infections	Cross-sectional	Going through all the data reported in the SINAN (i.e., used surveillance data)	Freshwater stingrays (genera of *Potamotrygon, Paratrygon, Plesiotrygon*, and *Heliotrygon*)	476 stingray injuries recorded 1.7 cases per 100,000 person/year Alvares (77.2 cases/100,000) Uarini (51.5 cases/100,000) Silves (20.4 cases/100,000)
Epidemiology of aquatic animal poisonings reported to a Colombian toxicology control center	Montoya DV et al., 2019 [76]	Antioquia, Colombia	2016-2018	Characterize the cases of envenoming by marine and freshwater animals in Colombia	Cross-sectional retrospective descriptive study	Data was extracted from the Poison control center (PCC) of the University of Antioquia, Colombia. Cases identified as aquatic animal envenomations were called as a follow-up to determine if any complications or long-term sequelae occurred.	Aquatic animals Freshwater stingray Cnidaria	12 (1.2%) 11 (1.1%) 1 (0.1%)
Injuries caused by fish to fishermen in the Vale do Alto Jurua, Western Brazilian Amazon	Costa TND et al., 2020 [77]	Cruzeiro do Sul (Acre State, Brazil)	2017	Determine clinical, epidemiological and therapeutic aspects of injuries caused by fish among professional fishermen	Cross-sectional retrospective descriptive analysis	Interviews with fishermen from the Z1 fishermen community and the Resende de Souza Lima fish market	Stingray (“*arraia*”, *Potamotrygon* sp.) Catfish (“*mandi*”, *Pimelodus* spp.) Other pimelodid fishes	20 (9.8%) 108 (52.9%) 20 (9.8%)
Mortality caused by venomous animals in Venezuela (2000-2009): A new epidemiological pattern	De Sousa L et al., 2021 [78]	Venezuela	2000-2009	Assess mortalities resulting from contact with venomous animals in Venezuela from 2000 to 2009	Cross-sectional	The information was extracted from the annual mortality records of the Venezuelan Ministry of Health. “Other” included X21, X25, X26, X27, and X29. Venomous marine animals and plants = X26	Other	84 (11.1%) **Year - Frequency - Incidence (/100,000)** 2000 - 13 (1.7%) - 0.05 2001 - 5 (0.7%) - 0.02 2002 - 10 (1.3%) - 0.04 2003 - 12 (1.6%) - 0.05 2004 - 7 (0.9%) - 0.03 2005 - 10 (1.3%) - 0.04 2006 - 1 (0.1%) - 0.004 2007 - 6 (0.8%) - 0.02 2008 - 13 (1.7%) - 0.05 2009 - 7 (0.9%) - 0.02
Temporal trend and epidemiological profile of accidents involving venomous animals in Brazil, 2007-2019	De Souza TC et al., 2022 [79]	Brazil	2007-2019	Analyze the temporal trends of accidents involving venomous animals in Brazil	Cross-sectional	Information was extracted from TABNET SINAN between 2007 to 2019 involving venomous animal accidents. “Other” includes Hymenoptera, beetles, centipedes, fish, and cnidaria	Other	87,231 (4.1%) Deaths 64 (0.07% case fatality ratio) Incidence 3.8 cases/100,000
Venomous animals in Pernambuco: children at risk	Albuquerque MCD et al., 2022 [80]	Pernambuco, Brazil	2017-2019	Analyze the epidemiological and clinical aspects caused by venomous animals in children under 15 years old	Cross-sectional	Used secondary data from a Poison Center in Pernambuco (Centro de Informação e Assistência Toxicológica de Pernambuco - CIATox-PE)	Aquatic animals	19 (0.7%)
Europe								
Relationships among injuries treated in an emergency department that are caused by different kinds of animals: epidemiological features	Massari M and Masini L, 2006 [81]	Pesaro and small municipalities around it, Central Italy	1998-1999	Analyze animal-related injury features treated in an emergency department	Cross-sectional	All patients treated in the ED for an injury caused by an animal, over a 2-year period. Inspection of all reports concerning animal-related injuries. Each report inspected individually as code-based diagnosis research was not available	Marine fauna: Jellyfish Weever fish Sea urchin Scorpion fish Sea anemone Urticant animal	73 (7.4% AI, 0.123% ED) 18 (1.82% AI, 0.030% ED) 30 (3% AI, 0.050% ED) 13 (1.32% AI, 0.020% ED) 1 (0.1% AI, 0.002% ED) 9 (0.9% AI, 0.020% ED) 1 (0.1% AI, 0.002% ED)
Impact of stinging jellyfish proliferations along South Italian Coasts: human health hazards, treatment and social costs	De Donno A et al., 2014 [82]	Salento, Southern Italy	2007-2011	Investigate the epidemiology, severity and treatment of jellyfish stings over summer seasons across 5 years	Cross-sectional	Collection and analysis of data from patients registered yearly at medical first aid stations - 2 emergency ambulances, four hospitals, and 21 first-aid centers	Jellyfish	1733 (0.2%)
Marine envenomations in returning French travelers seen in a tropical diseases unit, 2008-2013	Henn A et al., 2016 [83]	Paris, France	2008-2013	Evaluate the prevalence and characteristics of marine envenomations in returning French travelers	Cross-sectional retrospective descriptive analysis	Medical chart analysis of all returning travelers presenting with a health problem om a French tropical disease unit - focus on marine envenomation. Returning travelers from: Oceania (4) Caribbean (4) Latin America (3) Asia (14) (SE Asia = 10) Africa (9) Europe (3)	Jellyfish Stonefish Corals Miscellaneous	8 (21.6% ME, 0.24% TDU) 10 (27.0% ME, 0.3% TDU) 11 (29.7% ME, 0.33% TDU) 8 (21.6% ME, 0.24% TDU)
Lifeguard assistance at Spanish Mediterranean beaches: jellyfish prevail and proposals for improving risk management	Bordehore C et al., 2016 [84]	Spanish Mediterranean beaches, Spain	2012	To analyze the nature of injuries from lifeguard-recorded data for the Spanish Mediterranean beaches for 2012 Facilitate precautionary management and reduce injuries based on a real-time beach assistance database of injuries to identify high incidence assistance categories	Cross-sectional retrospective descriptive study	The authors tried for census, that is, all cities with lifeguards. It is mandatory for cities to provide lifeguard services, at least during bathing season. They had a response rate of (760/1200) = 63.3% The authors called and emailed each responsible department of the coastal cities, as well as the city councils to gather information Responses classified as: good data, incomplete data due to lack of proper jellyfish sting data, incomplete data due to a partial or total lack of categories other than jellyfish, and no data	Jellyfish (presumptive) Jellyfish (reported) Other marine animal stings Sea Urchin Weever fish	116,887 (66.4%) 94,453 (53.7%) 22,434 (12.7%) 1197 (0.68%) 1980 (1.12%)
			2008-2023	Study any trend in jellyfish stings from the earliest summer available until 2012 using a Sting Index		For the Sting Index: the authors standardized reports by calculating the sum of all injuries other than jellyfish stings ("density-dependent" injuries) and dividing the total number of jellyfish stings by the sum	Jellyfish stings	2008 = 13,378 (71%) 2009 = 18,085 (57%) 2010 = 50,753 (71%) 2011 = 55,679 (67%) 2012 = 89,245 (72%)
Epidemiology of jellyfish stings using the Sting Index to identify trends and support proactive management	Dobson JY et al., 2024 [85]	Spanish Mediterranean beaches, Spain	2008-2022	This research is an expansion on the work initiated by Bordehore et al. [84] - to identify sting trends along Spanish beaches and quantify their effects on public health and coastal tourism	Cross-sectional retrospective descriptive study	Emailed city councils, mayors, environmental delegates and sent out follow-ups four months later to any non-respondents. Responses were categorized as "Good data", "Incomplete data" and "No data". Contacted 196 cities, 148 of these participated, which is 75.5% response rate. There are 211 municipalities total, so 70.1% were represented in this study. This included data from 787 lifeguard stations. Sting index: jellyfish sting numbers relative to a factor proportional to the number of beachgoers.	Jellyfish stings Other marine animal stings Sea urchins Weever fish and related Total aquatic	359,909 (54.5%) 31,501 (4.8%) 6140 (0.9%) 14786 (2.2%) 412,336 (62.4%)
Asia								
Poisoning in Israel: Annual Report of the Israel Poison Information Center, 2007	Bentur Y et al., 2008 [86]	Israel	2007	To analyze data on the epidemiology of poisonings and poison exposure in Israel	Cross-sectional	Computerized queries and descriptive analysis of medical records database from IPIC	Total aquatic Jellyfish Sea urchin Fish Other aquatic animals	112 (10.9% ABS, 0.42% TC) 22 (2.13% ABS, 0.08% TC) 4 (0.39% ABS, 0.015% TC) 74 (7.15% ABS, 0.28% TC) 12 (1.2% ABS, 0.04% TC)
Venomous fish injuries along the Israeli Mediterranean coast: scope and characterization	Gweta S et al., 2008 [87]	Mediterranean coast, Israel	2003-2004	To characterize and assess the extent of injuries caused by marine organisms along the Mediterranean coast of Israel. Their type, severity and medical treatment given	Cross-sectional	Random selection of fishermen, fishing anchorage, and interview days. Survey of professional fishermen who had sustained an injury from a marine organism	Jellyfish Stingrays Weever fish Rabbit fish Striped-sea catfish Scorpionfish Fire worms	1 (1.3%) 24 (30.4%) 17 (21.5%) 10 (12.7%) 8 (10.1%) 2 (2.5%) 1 (1.3%)
			1994-2004		Cross-sectional retrospective	All data that was in “biological” and “aquatic” categories between 2003 to 2004, and queries of "offending marine creatures" between 1997-2004, were retrieved from the database. And a medical chart review of people who called the Israel Poison Information Center. Total IPIC calls = 162,739	Total aquatic Jellyfish Fish Unknown	1188 (0.73%) 295 (24.8% MI) 730 (61.4%. MI) 163 (13.7% MI)
Environmental factors associated with the prevalence of animal bites or stings in patients admitted to an emergency department	Hsiao M-H et al., 2012 [88]	Taiwan	2007-2008	Present region-specific demographics of animal bites in central Taiwan	Prospective study	Starting in 2007, prospectively collected data on animal bites treated in the hospital's emergency department until the end of the study. Data was collected by trained individuals using a standardized data abstraction form	Jellyfish	2 (0.34% ABS, 0.001% ED)
Poisoning in Israel: annual report of the Israel Poison Information Center, 2012	Bentur Y et al., 2014 [89]	Israel	2012	To report data on the epidemiology of poisonings and poison exposure in Israel	Cross-sectional retrospective descriptive analysis	Case records in the database are from self-reported calls to the IPIC. All data entered and stored in a designated tailored database using Access 2007 on SQL server	Total Aquatic Jellyfish Fish Other aquatic animals	75 (4.35% BA, 0.24% PEC) 7 (0.4% BA, 0.02% PEC) 47 (2.72% BA, 0.15% PEC) 21 (1.22% BA, 0.07% PEC)
Trapped in a sea of uncertainty: limitations in unintentional injury research in the Philippines and interdisciplinary solutions to reduce fatal box jellyfish stings	Pirkle C and Yanagihara AA, 2019 [36]	Coastal regions of Philippines	2018	Obtain data about box jellyfish stings in communities and provide information about current approaches to sting victims. Aims to improve first-aid recommendations and guidelines	Cross-sectional	10-minute survey to attendees (convenience sample) of a workshop about jellyfish stings	Box jellyfish	17 (32%)
Poisoning in Israel: Annual Report of the Israel Poison Information Center, 2017	Bentur Y et al., 2019 [90]	Israel	2017	To report the epidemiology of poison exposures in Israel	Cross-sectional retrospective descriptive analysis	Computerized queries and a descriptive analysis of the medical records database of IPIC	Total aquatic Jellyfish Catfish Sea urchin Fish Other/unknown	62 (4.12% BA, 0.16% PEC) 25 (1.66% BA, 0.06% PEC) 6 (0.4% BA, 0.02% PEC) 3 (0.2% BA, 0.01% PEC) 16 (1.06% BA, 0.04% PEC) 12 (0.8% BA, 0.03% PEC)
Africa								
South African marine envenomations and poisonings as managed telephonically by the Tygerberg Poisons Information Centre: a 20-Year retrospective review	Marks CJ et al., 2019 [91]	South Africa	1995-2014	Epidemiological review of marine toxicity	Cross-sectional retrospective descriptive analysis	Retrospective analysis of consultation forms that were transcribed from 311 calls to the Tygerberg Poisons Info Center	Marine ENV Bluebottle Jellyfish Stingray Sea barbel Scorpionfish Sea anemone Sea urchin Starfish	153 (0.18%) 33 (0.04%) 14 (0.02%) 36 (0.04%) 30 (0.04%) 14 (0.02%) 1 (0.001%) 10 (0.01%) 2 (0.002%)
Epidemiology of the cnidarian *Pelagia noctiluca* stings on Moroccan Mediterranean beaches	Mghili B et al., 2020 [92]	Morocco	2018	An epidemiological study on the stings of *Pelagia noctiluca*	Cross-sectional	Data retrieved from patients seeking medical treatment after jellyfish sting.	Jellyfish	1321 (70%)
Tropical marine faunal hazard knowledge, incidents and associated health burden among seascape users at the Kenyan coastline	Kihia CM et al., 2023 [93]	Kenya	Does not report	Document indigenous knowledge of harmful marine biota and incidence of envenomation and estimate health cost and associated financial burden	Cross-sectional	Structured questionnaire interviews with seascape users in Mtwapa and Gazi, Kenya, including boat fishers, beach traders, foot fishers, and beach boys/girls. Community members were also included, following snowball sampling from seascape user interviewees. Information collected included the types of marine organisms causing injury and envenoming, symptoms and incident frequency.	Urchins Stingray Catfish	Incidents/year 142.31 141.21 4.2
Oceania								
An analysis of marine animal injuries presenting to emergency departments in Victoria, Australia	Taylor DM et al., 2002 [41]	Victoria, Australia	1995-2000	Describe the epidemiology of marine animal injuries in Victoria, Australia, to identify risk factors and recommend prevention strategies	Cross-sectional. retrospective, descriptive study	Chart review of presumably all marine animal injury cases seeking ED care from 22 locations. Data was obtained from Victorian Emergency Minimum Dataset, which contains patient demographic and injury details. Doctors, nurses or clerks enter these data into the electronic fields. Searched the following key terms within the VEMD "description of injury event": stingray, fish, shark, eel, jellyfish, jelly fish, crayfish, cray fish, crab, octopus, urchin, coral, anemone, shell, shellfish, shell fish, lice	Total aquatic Jellyfish Stingrays Sea urchin Coral Fish	181 (88.3% MI, 0.022% ED) 42 (20.5% MI, 0.005% ED) 46 (22.4% MI, 0.006% ED) 7 (3.4% MI, 0.0009% ED) 3 (1.5% MI, 0.0004% ED) 83 (40.5% MI, 0.01% ED)
Leisure-related injuries at the beach: an analysis of lifeguard incident report forms in New Zealand, 2007-2012	Moran K and Webber J, 2014 [94]	New Zealand	2007-2012	To describe the etiology of non-drowning related injuries occurring at surf beaches (patrolled by lifeguards)	Cross-sectional retrospective descriptive study	Presumably all national incident report forms (SLSNZ) excluding drowning-related, those sustained in a non-leisure activity (employment), and those with insufficient information. It is unclear if all forms were reviewed.	Jellyfish	1376 (16.29%)
Injury trends from envenoming in Australia, 2000-2013	Welton RE et al., 2017 [95]	Australia	2000-2013	Provide the first contemporary epidemiological insight into venomous injuries based on demographics and geographic nationally in Australia	Cross-sectional	Hospitalized cases in the Australian Institute of Health and Welfare (AIHW) and National Coronial Information System (NCIS) were examined. The authors also used medical literature and media reports - cases from minimum of two sources were included	Venomous marine animals and plants Fish Other Jellyfish (death)	3707 (9.36% ENV) 424 (1.21% ENV) 2188 (6.24% ENV) 3 (5%)
Environmental deaths in the northern territory of Australia, 2003-2018	Tiemensma M, 2019 [96]	Northern Territory of Australia	2003-2018	To describe the environmental deaths occurring in the Northern Territory of Australia	Cross-sectional retrospective descriptive study	All cases reported to the NT coroner office and Royal Darwin Hospital Forensic Pathology Unit as environmental deaths were analyzed	Jellyfish (*Chironex fleckeri*)	1 (0.02% TC, 0.6% Enviro)
Animal bite wounds and their management in tropical Australia	Vardanega J et al., 2022 [97]	Cairns, Australia	2013-2020	To define the microbiologic characteristics of animal bites in tropical Australia and the appropriateness of current Australian antimicrobial guidelines for their management. To inform and optimize management strategies	Cross-sectional retrospective audit examining hospitalizations records in tropical Australia	Cairns Hospital > 531 beds serving population of approx. 280,000 people and is a tertiary referral center for surrounding rural and regional hospitals. Hospitalizations with a completed discharge summary were included, electronical medical records examined	Total aquatic Jellyfish Fish Stonefish Stingrays Sea urchin Sea snake	211 (11.47%) 129 (7%) 35 (2%) 22 (1%) 17 (1%) 5 (0.3%) 3 (0.17%)
Australian sea snake envenoming causes myotoxicity and non-specific systemic symptoms - Australian Snakebite Project (ASP-24)	Johnston C et al., 2022 [98]	Australia	2002-2020	We aimed to describe the epidemiology and clinical presentation of Australian sea snake envenoming and the effectiveness of antivenom	Cross-sectional Prospective observational study	Patients were recruited to the Australian Snakebite Project (ASP), an Australia-wide prospective observational study recruiting all patients with suspected or confirmed snakebite >2 years. Information about demographics, bite circumstances, species involved, clinical and laboratory features of envenoming, and treatment is collected and entered a purpose-built database	Sea snake	13 (0.6%)

Characteristics of fifty-three articles included in this review. ENV: envenomation; TC: total cases; UCD: underlying cause of death; MCD: multiple causes of death; AQ ENV: aquatic envenomation; ABS: animal bites and stings; AI: animal injury; MI: marine injury; ED: emergency department visits; ME: marine envenomation; TDU: tropical disease unit visits; BA: biologic agent exposure; PEC: poison exposure cases.

### Geographic distribution of studies

**Figure 2.**
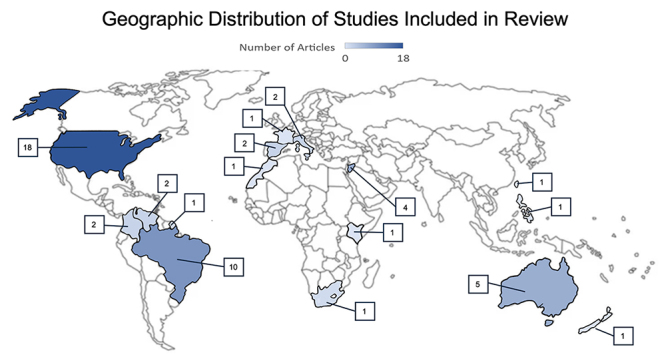
Geographic distribution of studies indicating their number per country.

Most studies (62%) were conducted in the Americas, specifically the United States in North America (18 articles), and Brazil (10 articles), Venezuela (2 articles), French Guiana (1 article), and Colombia (2 articles) in South America. The remaining articles described research in Europe (5), Asia (6) and Oceania (6). There were three articles from the African continent (South Africa, Kenya, and Morocco). The articles in Europe focused on regions in Italy and Spain or returning French travelers that had encountered marine envenomation while abroad. The articles describing studies within Asia were from Israel (4), the Philippines (1) and Taiwan (1). From Oceania, research from five articles was conducted in Australia and there was a single article from New Zealand (Figure 2).

### Envenomation prevalence

**Figure 3.**
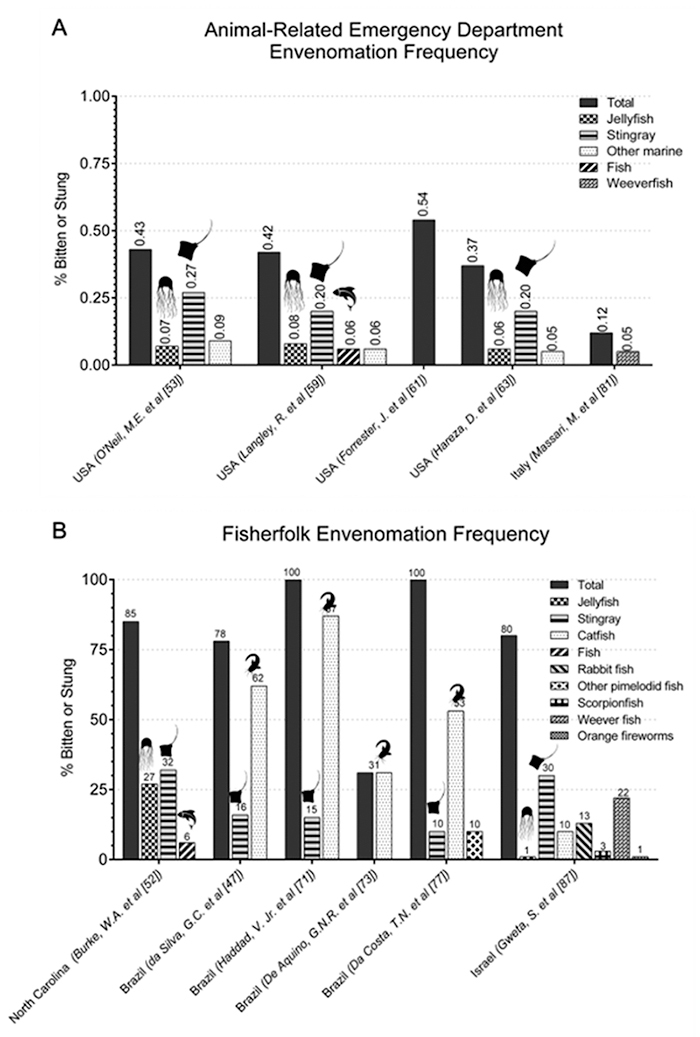
(A) Animal-related emergency department envenomation frequency. Only envenomation frequency values ≥ 0.05% were included to improve the readability of the figure; additional values below this cut-off can be found in Table 2. (B) Fisherfolk envenomation frequency. *In some studies, the total percentage of the population bitten or stung was 100%. This is because the entire sample (fishermen interviewed) had experienced a bite or sting in their life.

Envenomation events varied dramatically across studies. In some, especially those using emergency department (ED) visit data, well under 1% of the population studied experienced a marine envenomation (Figure 3A) [41, 53, 59, 61, 63, 81, 88]. Similarly, the population experiencing aquatic envenomation as described by total poison control and toxicology calls ranged from 0.01% to 0.73% (Additional file 2), but varied between 0.3% to just over 10% in terms of total envenomations reported (Additional file 3) [51, 54, 55, 57, 58, 60, 65-67, 70, 76, 80, 86, 87, 89-91]. In other populations, such as fisherfolk, numbers frequently exceeded 70% (Figure 3B) [47, 52, 71, 73, 77, 87].

In the USA, aside from the occupational exposure of fisherfolk [52], the bite or sting incidence from venomous marine animals was at or below 1% in terms of overall animal-related fatalities, or envenomation via toxico-surveillance [50, 51, 53-67]. Four articles in the USA reported a total of five deaths by “venomous marine animals and plants” or by “fish stings” but did not identify the exact species responsible [50, 56, 60, 62].

Most of the South American reports originated from Brazil. The majority of these were interviews with selected high-risk populations including fishermen (3 articles) or riverside dwelling peoples (3 articles) [47, 68, 71, 73, 74, 77]. Studies of envenomation incidents in these population groups predominantly documented stings by catfish and stingrays. Three articles from Brazil used SINAN to quantify accidents caused by venomous animals [79], or accidents specifically caused by aquatic animals [40], or risk factors associated with freshwater stingray injuries [75]. Stingray envenomation accounted for 69% of aquatic animal injury [40] and caused 1.7 sting cases per 100,000 persons/year [75]*.* Mortality caused by venomous animals was reported in two articles in Venezuela, via their National Health System [72, 78]. However, similar to the mortality data from the USA, venomous marine animals and plants were not specifically identified, and further grouped with other categories of “unspecified animals”, resulting in a total of 58 and 84 deaths, but not indicating the direct cause [72, 78]. Telephone reports to emergency services in French Guiana and Colombia due to cnidarians or aquatic animals represented 0.005% and 0.07% of total calls made, respectively [69, 70]. This represented 1.5% of total envenomations in Colombia during the study period (2006 to 2010) [70]; between 2016 and 2018, aquatic envenomations were responsible for 1.2% of the total envenomations reported to the poison control center of the University of Antioquia, Colombia [76].

Similar to the USA, three articles from Europe reported less than 1% of admissions to ED’s, medical first aid stations, and a tropical disease unit were due to marine fauna including venomous species [81-83]. However, in an analysis of injuries documented by lifeguard reports along Spanish Mediterranean beaches, 66% of incidents were due to jellyfish envenomation [84]. An extension of this study, spanning 15 years, demonstrated that aquatic envenomations, and jellyfish stings predominantly, were consistently responsible for approximately 62% of injuries reported to lifeguards [85].

Three articles from Asia (including the Middle East) described annual reports from the Israel Poison Information Center, indicating venomous aquatic animals caused below 0.5% of total reported poison exposure cases, annually [86, 89, 90]. A study from Taiwan investigated cases of patients presenting with animal bites or stings in the ED, of which 0.34% were due to jellyfish [88]. Finally, two community-based interviews along the Israeli Mediterranean coast and in the Philippines reported 81% of fishermen and 32% of community members (health practitioners, government employees and military/police) respectively, had experienced envenomation [36, 87]. The study from Australia using data from ED visits reported similar frequencies of envenomations as those reported in the USA and Europe (less than 0.1% of all deaths) [41]. When examining envenomation or animal bite and/or stings, between 7-11% of the cases were due to venomous marine animals and plants [95, 97]. Sea snake bites accounted for 0.6% of all snakebites in an Australian-wide prospective study [98]. Finally, jellyfish envenomation caused 16% of leisure-related injuries at beaches in New Zealand [94]. The reports from Africa came from diverse sources - a poison information center in South Africa, first-aid stations along four Mediterranean beaches in Morocco, and seascape user reports from Mtwapa and Gazi, Kenya [91, 92, 93]. Marine envenomations accounted for 0.18% of consultations at the Tygerberg poisons information center (South Africa) [91] but 70% of visits to Moroccan beach first-aid stations [92]. Seascape users in Kenya reported approximately 140 injury incidents per year by both urchins and stingrays [93]. 

### Study characteristics of articles describing aquatic envenomation

As shown in Table 2, the study design of nearly all articles (96%) was cross-sectional, either by administering surveys directly to specific populations, or analyzing reports from various databases including ED visits, calls to poison control centers, a notifiable disease database (SINAN, Brazil), or from coroner offices and mortality databases (CDC Wonder). There were 17 articles that reported data from within a single year, while 23 articles (43%) reported data from a period of 2-9 years. Fewer articles (17%) included longer study periods (10-20 years).

Of the 18 envenomating organisms included among our search terms, 14 were described by articles in this review. The most common venomous organisms described were jellyfish (37), including five articles mentioning box jellyfish and six describing Portuguese man-of-war or *Physalia*, stingrays (18), sea urchins (13) and catfish (11). Of the ten articles that included catfish envenomation, 45% were from a single research group in Brazil. Several of the poison control and fatality databases did not provide data on specific organisms, but instead generalized to “fish stings”, “venomous marine animals” or “venomous marine animals and plants”.

### Prevalence of envenomations by individual socio-demographic characteristics

Based on the PRISMA guidelines for equity reporting and using the PROGRESS-Plus framework, Table 3 compares the percentages of bites/stings experienced by different population groups, such as men and women or by race/ethnicity [36, 40, 41, 47, 50, 51, 52, 54-56, 60-62, 64-68, 71-83, 87, 92, 93, 95, 97, 98].

**Table 3.** Envenomation by socio-demographic characteristics.

Title	Author, year published, ref.	Organisms	Bites or stings by age (years)	Bites or stings by sex/gender	Bites or stings by race/ethnicity	Bites or stings by occupation
North America						
Animal-related fatalities in the United States - an update	Langley RL, 2005 [50]	Venomous marine animals	0-4 (0, 0%) 5-9 (0, 0%) 10-19 (0, 0%) 20-64 (1, 50%) > 65 (1, 50%)	M (2, 100%)	White (2, 100%)	
Pattern of stingray injuries reported to Texas poison centers from 1998 to 2004	Forrester MB, 2005 [51]	Stingray	<6 (3, 2%) 6-19 (36, 25%) >19 (107, 73%)	M (115, 75%) F (37, 24%)		
Skin problems related to the occupation of commercial fishing in North Carolina	Burke WA et al., 2006 [52]	Jellyfish, stingrays, spiny dogfish, and sea catfish	24-79 (Mean 52)	M (81, 100%)		Fishermen (81, 100%)
Animal bites and stings reported by United States poison control centers, 2001-2005	Langley RL, 2008 [54]	Coelenterate, fish, and other/ unknown	< 6 (13%) 6-19 (29% total, 50% of Coelenterate stings) 19+ (57%)			
2011 Annual Report of the American Association of Poison Control Centers' National Poison Data System (NPDS): 29th Annual Report	Bronstein AC et al., 2012 [55]	Fish stings	< 5 (25, 3%) 6-12 (53, 6%) 13-19 (88, 10%) > 20 (624, 70%) Unknown (98, 11%)			
		Cnidaria	< 5 (69, 13%) 6-12 (145, 27%) 13-19 (100, 19%) > 20 (184, 34%) Unknown (41,8%)			
		Other/unknown	< 5 (189, 55%) 6-12 (31, 9%) 13-19 (20, 6%) > 20 (77, 23%) Unknown (24, 7%)			
Fatalities from venomous and nonvenomous animals in the United States (1999-2007)	Forrester JA et al., 2012 [56]	Venomous marine animals and plants	35-64 (1, 100%)	M (1, 100%)	Black (1, 100%)	
2015 Annual Report of the American Association of Poison Control Center -National Poison Data System (NPDS): 33rd Annual Report	Mowry JB et al., 2016 [60]	Fish stings	< 5 (25, 4%) 6-12 (32, 5.2%) 13-19 (62, 10%) > 20 (450, 72.7%) Unknown (50, 8%)			
		Cnidaria	< 5 (45, 13.8%) 6-12 (63, 19.3%) 13-19 (56, 17%) > 20 (130, 39.8%) Unknown (33, 10.1%)			
		Other/unknown	< 5 (157, 57.3%) 6-12 (22, 8%) 13-19 (11, 4%) > 20 (65, 24%) Unknown (19, 7%)			
Mortality, hospital admission, and healthcare cost due to injury from venomous and non-venomous animal encounters in the USA: 5-year analysis of the National Emergency Department Sample	Forrester JD et al., 2018 [61]	Venomous marine animals and plants	0-17 (9625, 28%) 18-44 (16684, 48%) 45-64 (7059, 20%) 65-74 (1283, 4%) 75-84 (205, 1%) > 85 (15, 0%)	M (20664, 59%) F (14359, 41%)		
Animal-Encounter Fatalities: United States, 1999-2016: Cause of Death and Misreporting	Haskell MG and Langley RL, 2020 [62]	Venomous marine animals and plants	UCD: 35-64 (1, 100%) MCD: 35-64 (1, 50%) 65+ (1, 50%)			
Envenomations during pregnancy reported to the national poison data system, 2009-2018	Ramirez-Cruz MP et al., 2020 [64]	Fish stings, jellyfish and coelenterates, and other	15-44 (2134, 100%)	F (2139, 100%)		
2020 Annual Report of the National Poison Data System© (NPDS) from America's Poison Centers: 38th Annual Report	Gummin DD et al,. 2021 [65]	Fish stings	< 5 (15, 2.4%) 6-12 (41, 6.6%) 13-19 (67, 11%) > 20 (444, 72%) Unknown (53, 9%)			
		Cnidaria	< 5 (37, 14%) 6-12 (75, 28%) 13-19 (44, 17%) > 20 (91, 34%) Unknown (18, 7%)			
		Other/Unknown	< 5 (299, 54%) 6-12 (42, 8%) 13-19 (32, 6%) > 20 (143, 26%) Unknown (38, 7%)			
2021 Annual Report of the National Poison Data System© (NPDS) from America's Poison Centers: 39th Annual Report	Gummin DD et al., 2022 [66]	Fish stings	< 5 (22, 4.5%) 6-12 (30, 6.1%) 13-19 (45, 9.2%) > 20 (354, 72.5%) Unknown (37, 7.6%)			
		Cnidaria	<5 (39, 15.1%) 6-12 (65, 15.2%) 13-19 (40, 15.4%) >20 (99, 38.2%) Unknown (16, 6.2%)			
		Other/Unknown	<5 (256, 51.1%) 6-12 (45, 9.0%) 13-19 (27, 5.4%) >20 (146, 29.1%) Unknown (27, 5.4%)			
Nationwide Aquatic Envenomations Reported to US Poison Control Centers from 2011 to 2020	Kirchberg TN et al., 2024 [67]	Aquatic envenomation	0-10 (1112, 14.7%) 11-20 (1545, 20.45) 21-30 (1607, 21.2%) 31-40 (1161, 15.3%) 41-50 (976, 12.9%) 51-60 (768, 10,1%) > 60 (417, 5.5%)	M (5243, 61.6%) F (3220, 37.8%)		
South America						
Puncture wounds by driftwood catfish during bucket baths: local habits of riverside people and fish natural history in the Amazon	Sazima I et al., 2005 [68]	Catfish	25-60 (27, 100%)	M (18, 67%) F (9, 33%)		
Injuries and envenomings by aquatic animals in fishermen of Coxim and Corumbá municipalities, State of Mato Grosso do Sul, Brazil: Identification of the causative agents, clinical aspects and first aid measures	Silva GCet al., 2010 [47]	Catfish, stingrays				Fishermen (100, 100%)
Trauma and envenoming caused by stingrays and other fish in a fishing community in Pontal do Paranapanema, State of São Paulo, Brazil: epidemiology, clinical aspects, and therapeutic and preventive measures	Haddad Junior Vet al., 2012 [71]	Catfish, stingrays				Fishermen (39, 100%)
Mortality caused by venomous animals in Venezuela: 1980-1999	De Sousa L et al., 2014 [72]	“Other” including venomous marine animals and plants	0-9 (12, 21%) 10-19 (5, 9%) 20-29 (16, 28%) 30-39 (5, 9%) 40-49 (5, 9%) 50-59 (4, 7%) 60-69(4, 7%) > 70 (7, 12%)	M (41, 71%) F (17, 29%)		
Injuries caused by aquatic animals in Brazil: an analysis of the data present in the information system for notifiable diseases	Reckziegel GCet al., 2015 [40]	All venomous aquatic animals	< 9 (391, 10%) 10-19 (999, 24%) 20-34 (1327, 32%) 35-49 (909, 22%) 50-64 (401, 10%) > 65 (90, 2%) Not specified (1, 0%)	M (3146, 76%) F (972, 24%)		
		Stingrays	< 9 (158, 6%) 10-19 (649, 23%) 20-34 (986, 35%) 35-49 (693, 24%) 50-64 (291, 10%) > 65 (65, 2%)	M (2308, 81%) F (534, 19%)		
		Jellyfish	< 9 (193, 36%) 10-19 (179, 33%) 20-34 (108, 20%) 35-49 (43, 8%) 50-64 (14, 3%) > 65 (3, 1%)	M (270, 50%) F (270, 50%)		
		Toadfish	< 9 (5, 3%) 10-19 (28, 15%) 20-34 (55, 30%) 35-49 (59, 32%) 50-64 (22, 12%) > 65 (11, 6%) Not specified (1, 1%)	M (140, 77%) F (41, 23%)		
		Catfish	< 9 (1, 1%) 10-19 (18, 25%) 20-34 (21, 30%) 35-49 (17, 24%) 50-64 (13, 18%) > 65 (1, 1%)	M (58, 82%) F (13, 18%)		
		Sea urchins	< 9 (2, 12%) 10-19 (5, 29%) 20-34 (8, 47%) 35-49 (1, 6%) 50-64 (1, 6%) > 65 (0, 0%) Not specified (0, 0%)	M (12, 71%) F (5, 29%)		
		Other	< 9 (32, 7%) 10-19 (120, 26%) 20-34 (149, 32%) 35-49 (96, 21%) 50-64 (60, 13%) > 65 (10, 2%)	M (358, 77%) F (109, 23%)		
Injuries caused by the venomous catfish “*pintado*” and “*cachara*” (*Pseudopltaystoma genus*) in fishermen of the Pantanal region in Brazil	Aquino GN et al., 2016 [73]	Catfish				Fishermen (149, 100%)
Injuries caused by freshwater stingrays in the Tapajos River Basin: a clinical and sociodemographic study	Abati PAM et al., 2017 [74]	Stingrays	18-76 (19, 100%) 25-65 (17, 90%)	M (14, 74%) F (5, 26%)		Fishermen & farmers (19, 100%)
Delayed healthcare and secondary infections following freshwater stingray injuries: risk factors for a poorly understood health issue in the Amazon	Sachett J et al., 2018 [75]	Freshwater stingray	0-10 (52, 11%) 11-20 (154, 32%) 21-30 (80, 17%) 31-40 (72, 15%) 41-50 (58, 12%) 51-60 (41, 9%) > 60 (19, 4%) (n = 476, 100%)	M (392, 82%) F (84, 18%) (n = 476, 100%)	Mixed (398, 85%) European (32, 7%) Indian (27, 6%) African (7, 2%) Asian (3, 1%) (n = 467, 98.1%)	Work related incident? Yes (118, 26%) No (334, 74%) (n = 452, 94.96%) Maintenance & Repair services (183, 57%) Farmer/fisher (126, 39%) Trade & service employee (6, 2%) Technician (5, 2%) Industry employee (2, 1%) (n = 323, 67.9%)
Epidemiology of aquatic animal poisonings reported to a Colombian toxicology control center	Montoya DV et al., 2019 [76]	Stingray and Cnidaria	0-10 (1, 9%) 11-20 (2, 18%) 21-30 (3, 27%) 31-40 (3, 27%) 41-60 (2, 18%)	M (7, 58.3%) F (5, 41.7%)		
Injuries caused by fish to fishermen in the Vale do Alto Jurua, Western Brazilian Amazon	Costa TNDet al., 2020 [77]	Stingray, catfish, other pimelodid fishes	20-77 (51, 100%)	M (51, 100%)		
Mortality caused by venomous animals in Venezuela (2000-2009): A new epidemiological pattern	De Sousa L et al., 2021 [78]	"Other” including venomous marine animals and plants	< 2 (8, 9.5%) 2-4 (1, 1.2%) 5-14 (7, 8.3%) 15-19 (5, 6%) 20-44 (25, 29.8%) 45-64 (21, 25%) > 65 (17, 20.2%)	M (64, 76%) F (20, 24%)		
Temporal trend and epidemiological profile of accidents involving venomous animals in Brazil, 2007-2019	De Souza TC et al., 2022 [79]	"Other" including hymenoptera, beetles, centipedes, fish, cnidaria	Children < 5 most affected	Incidence (/100,000) M (3.9) F (2.8) RR = 1.4		
Venomous animals in Pernambuco: children at risk	Albuquerque MCD et al., 2022 [80]	Aquatic animals	< 1 (1, 5.3%) 1-4 (4, 21%) 5-9 (5, 26%) 10-14 (9, 47%)	Not specified for aquatic animals - overall trend for all envenomation was 54% male		
Europe						
Relationships among injuries treated in an emergency department that are caused by different kinds of animals: epidemiological features	Massari M and Masini L, 2006 [81]	Weever fish, jellyfish, sea urchin, scorpion fish, sea anemone, urticant, mussel	10-70 (73, 100%) (Mean 30.6, Med 26)	M (45, 62%) F (28, 38%)		
		Weever fish	(Mean 31.8, Med 32.5)	M (17, 57%) F (13, 43%)		
		Jellyfish	(Mean 24.1, Med 23.5)	M (11, 61%) F (7, 39%)		
		Sea urchin		M (10, 77%) F (3, 23%)		
		Sea anemone		M (6, 67%) F (3, 33%)		
Impact of stinging jellyfish proliferations along South Italian Coasts: human health hazards, treatment and social costs	De Donno A et al., 2014 [82]	Jellyfish	1-10 (502, 29%) 11-20 (433, 25%) 21-30 (294, 17%) 31-40 (173, 10%) 41+ (346, 20%)	M (53%) F (47%)	Italian (1074, 62%) Italian tourists (624, 36%) Foreign tourists (52, 3%)	
Marine envenomations in returning French travelers seen in a tropical diseases unit, 2008-13.	Henn A et al., 2016 [83]	Corals	Med 38, Range 26-63	M (5, 45%) F (6, 55%)		Tourism (37, 100%)
		Stonefish	Med 42.5, Range 30-58	M (4, 40%) F (6, 60%)		
		Jellyfish	Med 57.5, Range 27-68	M (5, 63%) F (3, 37%)		
		Other	Med 39.5, Range 25-52	M (4, 50%) F (4, 50%)		
Asia						
Venomous fish injuries along the Israeli Mediterranean coast: scope and characterization	Gweta Set al., 2008 [87]	Stingrays, Weever fish, Rabbit fish, Striped sea catfish, Scorpionfish, Jellyfish, Fire worms				Fishermen (79, 100%)
		All marine injuries	> 18y (76.1%) 13-18 (7.5%) 6-12 (6.9%) <6 (3.3%)			
Trapped in a Sea of Uncertainty: Limitations in Unintentional Injury Research in the Philippines and Interdisciplinary Solutions to Reduce Fatal Box Jellyfish Stings	Pirkle C and Yanagihara AA, 2019 [36]	Jellyfish	Mean 42, Range 31-53	M (14, 82%) F (3, 18%)		Health practitioner (4, 27%) Government employee (7, 47%) Military/Police (4, 27%)
Africa						
Epidemiology of the cnidarian *Pelagia noctiluca* stings on Moroccan Mediterranean beaches	Mghili B et al., 2020 [92]	Jellyfish	0-10 (~18%) 11-20 (515, 39%) 21-30 (330, 25%) 31-40 (~10%) 41-50 (~5%) 51-60 (~2%) 60+ (~1%)	M (881, 66%) F (440, 34%)	Moroccan (90%) Foreign visitors (9%)	
Tropical marine faunal hazard knowledge, incidents and associated health burden among seascape users at the Kenyan coastline	Kihia CM, et al. 2023 [93]	Lionfish, stonefish, stingray, urchin, jellyfish, sea snake, catfish				Occurrence rate (/yr) Beach trader (2.96 ± 12.31) Beach boy (0.66 ± 19.64) Foot fisher (11.73 ± 14.57) Boat fisher (4.23 ± 10.17)
Oceania						
An analysis of marine animal injuries presenting to emergency departments in Victoria, Australia.	Taylor DM et al., 2002 [41]	Jellyfish	5-29 (32, 76%)			
		Jellyfish, stingray, sea urchin, coral, fish	1-82 (Mean 29.5) 50+ (21, 10%)	M (147, 72%) F (57, 28%)		
Injury trends from envenoming in Australia, 2000-2013	Welton RE et al., 2017 [95]	Venomous marine animals and plants		M (2617, 71%) F (1090, 29%)		
		Toxic effect of contact with fish		M (345, 81%) F (79, 19%)		
		Toxic effect of contact with other marine animals		M (1378, 63%) F (810, 37%)		
Animal bite wounds and their management in tropical Australia	Vardanega Jet al., 2022 [97]	Jellyfish	(Median 23)	M (71, 55%) F (58, 45%)		
		Fish	(Median 36)	M (28, 80%) F (7, 20%)		
		Stonefish	(Median 33)	M (15, 68%) F (7, 32%)		
		Stingray	(Median 44)	M (15, 88%) F (2, 12%)		
Australian sea snake envenoming causes myotoxicity and non-specific systemic symptoms-Australian Snakebite Project (ASP-24)	Johnston C et al., 2022 [98]	Sea snake	0-10 (1, 7.7%) 11-20 (4, 30.8%) 21-30 (3, 23%) 31-40 (0) 41-50 (1, 7.7%) 51-60 (2, 15.4%) 60+ (2, 15.7%)	M (11, 84.6%) F (2, 15.4%)		Research (1, 7.7%) Fishing (8, 61.5%) Unknown (4, 30.8%)

Socio-demographic characteristics of those who experienced an aquatic venomous sting or bite. M = Male; F = Female.

**Figure 4.**
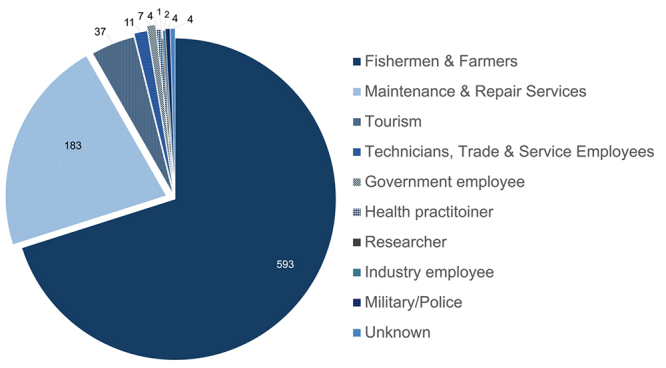
Occupation of those who experienced aquatic envenomation.

Thirty-seven reports characterized envenomation events by population characteristics (Table 3). The most commonly recorded groupings were age and sex. When both sexes were evaluated, the majority of envenomations occurred in men, aside from the study evaluating returned French travelers attending a tropical disease unit [83]. Envenomation by race/ethnicity was recorded in five articles [50, 56, 75, 82, 92]. When studies included information about occupation, the most affected group was fisherfolk (Figure 4) [47, 52, 71, 73, 74, 87]. However, multiple articles specifically selected and interviewed this group. Another study that recorded data on jellyfish envenomations from the Philippines, using a convenience sample, reported on events among participants of a series of health workshops in a high-risk region of the country. Envenomations were reported by health practitioners, government employees, and military police [36]. One article using SINAN to examine stingray injuries identified maintenance and repair technicians (57%), and farmers/fishermen (39%) as at-risk occupations [75]. Educational levels were recorded in two articles, both indicating that envenomation occurred more frequently among those with less education [75, 77]. For stingray envenomation, 43% of victims were illiterate or had less than four years of schooling [75]. Similarly, in another Brazilian article, 90% of those that experienced stingray, catfish or other pimelodid fish injury were illiterate or had incomplete elementary education [77]. Two studies from the USA used health insurance to proxy socioeconomic status. In the study assessing skin problems amongst commercial fishermen, envenomation appeared more common among those with health insurance [52]. The other study included ED patients’ insurance information as well as their household income as compared to their zip codes [61]. Envenomation was reported more in those with private-insurance (57%), followed by self-pay (18%) and Medicaid (14%) [61]. There was also a trend seen in terms of household income, where envenomation was more frequent among those in the highest income quartile relative to their zip code, compared to lower ones [61]. The authors did not test for statistical significance.

### Envenomation first aid and treatment

Very diverse first aid and treatment methods were reported, including alcohol (4 articles), gasoline (2), and smoke (3) (Table 4) [47, 64, 67, 68, 71, 73, 74, 76, 77, 82, 83, 87, 92, 97, 98]. The most common treatments were analgesics and heat/hot water. Analgesics were given in response to cnidaria, stingray, catfish, and stonefish envenomations [67, 76, 77, 82, 83, 87, 92]. They were commonly used in combination with antibiotics, corticosteroids, or antihistamines [67, 76, 77, 82, 83, 87, 92, 97, 98]. Hot water was indicated in seven articles for envenomations by the following species: catfish (3 articles), stingrays (5), and jellyfish (3) [47, 71, 73, 74, 77, 82, 87]. Hot water was also indicated as the preferred treatment for fish injuries among fishermen along the Israeli Mediterranean coast, including those by weever fish, rabbit fish, catfish, scorpionfish, stingrays, jellyfish, and orange fire worms [87]. Antivenom was used for one instance of stonefish and commonly for sea snake envenomation [83, 98]. Two articles mentioned that antivenom is not available for catfish or stingrays [73, 74], while the remaining studies did not report or mention antivenom at all. Surgery was reported in four studies, following stingray, stonefish and catfish envenomation (Table 4) [67, 68, 83, 97]. Compared to the other regions, management was described in five out of the six articles by the same research group in Brazil. Management was rarely described in the articles reporting envenomations treated in hospital settings or in reports to poison control centers.

**Table 4.** First-aid and treatments for aquatic envenomation

Title	Author, year published, ref.	Species	Antivenom	Antibiotics	Corticosteroids	Antihistamines	Analgesics	Surgery	Tied	Heat	Ice/cold	Urine	Alcohol	Coffee	Gasoline	Kerosene	Smoke	Fish parts	Salt/brine	Herbs	Blessings	Other
North America																						
Envenomations during pregnancy reported to the national poison data system, 2009-2018	Ramirez-Cruz MP, et al. 2020 [64]	Fish, jellyfish, other cnidaria, and unknown	N	3 (6%)																		
Nationwide Aquatic Envenomations Reported to US Poison Control Centers from 2011 to 2020	Kirchberg TN, et al. 2024 [67]	Fish, Cnidaria and Unknown	N	977 (11%)	459 (5%)	721 (8%)	31 (0.4%)	2 (0.02%)														Wash 4255, 50% IV 98, 1.2% Anti-nausea 33, 0.4% Benzodiazepines 22, 0.3% Sedation 10, 0.1% Oxygen 9, 0.1% Vasopressors 7, 0.1% Ventilator 5, 0.1% Intubation 5, 0.1% Bronchodilators 3, 0.04% Antihypertensives 3, 0.04% CPR 1, 0.01%
South America																						
Puncture wounds by driftwood catfish during bucket baths: local habits of riverside people and fish natural history in the Amazon	Sazima I, et al. 2005 [68]	Catfish (17)	N					1, 6%														
Injuries and envenomings by aquatic animals in fishermen of Coxim and Corumbá municipalities, State of Mato Grosso do Sul, Brazil: Identificaiton of the causative agents, clinical aspects and first aid measures	Silva GC, et al. 2010 [47]	Total (78)	N						4, 5%	10, 13%	1, 1%	3, 4%	15, 19%	Y	4, 5%	1, 1%	3, 4%	8, 10%	4, 5%	11, 14%	2, 3%	Other: Clay, sand, leaf litter, termite pupa or coffee powder 11, 14%
		Catfish (10)							1, 1%	3, 4%	1, 1%	1, 1%	5, 6%	Y	1, 1%	0	0	1, 1%	1, 1%	1, 1%	1, 1%	Other: Clay, sand, leaf litter, termite pupa or coffee powder 6, 8%
		Duckbill catfish (9)							3, 4%	0	0	1, 1%	1, 1%	Y	0	0	1, 1%	2, 3%	1, 1%	2, 3%	0	Other: Clay, sand, leaf litter, termite pupa or coffee powder 2, 3%
		Shovelnose catfish (3)							0	1, 1%	1, 1%	0	0	0	0	1, 1%	0	1, 1%	0	0	0	
		Spotted catfish (14)							0	3, 4%	0	1, 1%	7, 9%	Y	2, 3%	0	2, 3%	3, 4%	2, 3%	4, 5%	0	Other: Clay, sand, leaf litter, termite pupa or coffee powder 2, 3%
		Stingrays (7)							0	3, 4%	0	0	2, 3%	Y	1, 1%	0	0	1, 1%	0	4, 5%	1, 1%	Other: Clay, sand, leaf litter, termite pupa or coffee powder 1, 1%
Trauma and envenoming caused by stingrays and other fish in a fishing community in Pontal do Paranapanema, State of Sao Paulo, Brazil: epidemiology, clinical aspects, and therapeutic and preventive measures	Haddad Junior V, et al. 2012 [71]	Stingrays (6)	N							1, 17%		2, 33%	2, 33%				2, 33%	1, 17%		6, 100%		Contact with human vagina 1, 17%
Injuries caused by the venomous catfish pintado and cachara (Pseudopltaystoma genus) in fishermen of the Pantanal region in Brazil	Aquino GN, et al. 2016 [73]	Spotted and striped catfish	N							Y	Y	Y	Y		Y					Y		
Injuries caused by freshwater stingrays in the Tapajos River Basin: a clinical and sociodemographic study	Abati PAM, et al. 2017 [74]	Fresh-water stingray (19)	N							10, 53%												Homemade medication 10, 53% Avoid: Greasy food 17, 89% Chicken feces 16, 84% Sex 15, 79% Stepping on hot coals/earth 15, 79% Eye contact with pregnant women 13, 68%
Epidemiology of aquatic animal poisonings reported to a Colombian toxicology control center	Montoya DV, et al. 2019 [76]	Cnidaria (1)	N	1 (100%)			1 (100%)					1 (100%)										Sand 1, 100%
		Stingray (11)	N	6 (55%)			11 (100%)							Ash & coffee 1 (9%)								Chlorine 1, 9%
Injuries caused by fish to fishermen in the Vale do Alto Jurua, Western Brazilian Amazon	Costa TND, et al. 2020 [77]	Catfish (108)	N	1, 0.5%			1, 0.5%				1, 0.5%							1, 0.5%				Mercurochrome (medicine) 2, 1% Merthiolate 1, 0.5%
		Stingray (20)	N							Asphalt 1, 0.5% Compress 1, 0.5%				1, 0.5%			24, 12%					Acacu Hure crepitans milk 3, 1.5% Watermelon root 1, 0.5% Condensed milk 4, 2% Contact with human vagina 1, 0.5%
Europe																						
Impact of stinging jellyfish proliferations along South Italian Coasts: human health hazards, treatment and social costs	De Donno A, et al. 2014 [82]	Jellyfish (1733)	N		Local 797, 46% Systemic 745, 43%	Local 17, 1% Systemic 485, 28%	17, 1%			121-676, 7-39%	Cold water 52, 3% Ice 17, 1%		156, 9%									Liquid ammonia 1282, 74% Saline 208, 12% Chlorine-based disinfectant 121, 7%
Marine envenomations in returning French travellers seen in a tropical diseases unit, 2008-13.	Henn A, et al. 2016 [83]	Corals (11)	N	1, 9%	7, 64%	1, 9%																Anesthetic cream, valacyclovir 2, 18%
		Stonefish (10)	Y 3, 30%	5, 50%	1, 10%		2, 20%	Initial 1,10% At follow up 4, 40%														Local antifungal 1, 10%
		Jellyfish (8)	N	1, 13%	Topical 4, 50% Oral 1, 13%																	Ivermectin 1, 13%
		Miscellaneous (8)	N	3, 38%		2, 25%	4, 50%	Stingray = suture repair of a laceration injury 1, 12.5%														Non-steroid anti-inflammatory 2, 25%
Asia																						
Venomous fish injuries along the Israeli Mediterranean coast: scope and characterization	Gweta S, et al. 2008 [87]	Stingrays (24), Weever fish (17), Rabbit fish (10), Striped sea catfish (8), Scorpionfish (2), Jellyfish (1), Fire worms (1)	N				Y		Y	Y		Y				Y						No treatment Rinsing and bandaging Blood sucking Rest Burning cigarette on injury location Vinegar
		Jellyfish(295), Fish (730), Unknown (163)		Y	Y	Y	Y			Y												Cleansing and disinfection
Africa																						
Epidemiology of the cnidarian Pelagia noctiluca stings on Moroccan Mediterranean beaches	Mghili B, et al. 2020 [92]	Jellyfish	N	53, 4%	13, 1%		713, 54%				Y											Seawater rinse, applying sand Non-pharmacological treatments 542, 41%
Oceania																						
Animal bite wounds and their management in tropical Australia	Varda-nega J, et al. 2022 [97]	Jellyfish (129)	N	1, 0.78%				0														
		Stonefish (22)		10, 45%				2, 9%														
		Stingray (17)		16, 94%				8, 47%														Primary closure performed 4, 24%
Australian Sea Snake Envenoming Causes Myotoxicity and Non-Specific Systemic Symptoms-Australian Snakebite Project (ASP-24)	John-ston C, et al 2022 [98]	Sea snake	Y 8, 61.5%		1, 7.7%	2, 15.4%			10, 76.9%													

First-Aid and treatments received by those who experienced an aquatic venomous bite or sting. Y = Yes; N = No

Twenty-seven of the articles (51%) evaluated non-lethal outcomes and consequences associated with envenomation [40, 41, 47, 51, 52, 54, 55, 60, 61, 65-68, 71, 73-77, 82, 83, 87, 92, 93, 94, 97, 98]. Extremities (hands/feet) were the most common site of envenomation (Additional file 4) [40, 41, 68, 73, 75, 76, 82, 83, 87, 92, 94, 97, 98]. Stings by stingrays, stonefish, and sea urchin predominantly occurred on the feet, while stings by jellyfish did not have a consistent location [40, 41, 52, 82, 83, 92, 97]. Symptoms of envenomation were reported in 14 articles: pain (12 articles), edema (9), and erythema (9) were the most common (Additional file 5) [52, 68, 71, 73, 74, 75, 76, 77, 82, 83, 92, 93, 97, 98]. Other reported outcomes included shock (5 articles), pruritus (itchiness) (6) and secondary infection (8). By species, stingrays caused edema, pain, and the development of ulcers/lesions in the majority of populations studied [71, 74-77, 93]. Catfish envenomation caused pain for 50-100% of victims, reported in five articles [68, 73, 76, 77, 87]. Many symptoms were described in a single article each, including malaise, muscular spasms, wound soiling, and “non-specific systemic symptoms” [73, 82, 97, 98].

The time between envenomation and treatment was recorded in six studies (Table 5) [40, 75, 83, 87, 97, 98]. In the report investigating injuries caused by aquatic animals using SINAN, victims most often received treatment within 1 hour of envenomation, with the exception of sea urchin envenomation, in which it was more common to receive care after 24 hours [40]. In three additional studies, the time to treatment was within 0 to 5 hours [75, 87, 98]. Hospitalization was commonly reported following stingray envenomation; in three studies, 100% of those stung were hospitalized [71, 75, 97]. In contrast, only 16% and 4% of those injured by stingrays reported by a Texas poison center [51] and ED in Australia [41], respectively, were hospitalized. In a nationwide analysis of aquatic envenomation by cnidarians and fish reported to US poison control centers, only 13% of those stung were treated at a health care facility [67]. All of the thirteen sea snake envenomations reported by Johnston *et al.* [98] required hospitalization, and discharge most commonly occurred between 13-24 hours following admission.

**Table 5.** Morbidity and mortality

Title	Author, year published, ref.	Organism(s)	Elapsed time between accident to treatment	Hospitalization	Injury severity/disability	Death by bite
North America						
Animal-related fatalities in the United States - an update	Langley RL, 2005 [50]	Venomous marine animal				2, 0.1% (0.00067 deaths per million per year)
Pattern of stingray injuries reported to Texas poison centers from 1998 to 2004	Forrester MB, 2005 [51]	Stingray		Management site: On site 94, 61% En route to HCF 32, 21% Referred to HCF 25, 16% Unknown 2, 1%	No effect 0, 0% Minor effect 26, 53% Moderate effect 21, 43% Major effect 2, 4%	0
Animal bites and stings reported by United States poison control centers, 2001-2005	Langley RL, 2008 [54]	Coelenterates			No outcome 14, 1% Minor 387, 37% Moderate 72, 7% Major 1, 0.1%	0
		Fish			No outcome 12, 1% Minor 380, 29% Moderate 143, 11% Major 4, 0.3%	0
2011 Annual Report of the American Association of Poison Control Centers' National Poison Data System (NPDS): 29th Annual Report	Bronstein AC, et al. 2012 [55]	Fish stings		325, 37%	No effect (5, 0.6%) Minor (271, 31%) Moderate (107, 12%) Major (1, 0.1%)	0
		Cnidaria		114, 21%	No effect (6, 1%) Minor (177, 33%) Moderate (59, 11%) Major (1, 0.2%)	0
		Other/Unknown		55, 16%	No effect (48, 14%) Minor (35, 10%) Moderate (14, 4%) Major (3, 0.9%)	0
Fatalities from Venomous and Nonvenomous Animals in the United States (1999-2007)	Forrester JA, et al. 2012 [56]	Venomous marine animals and plants				1, 0.1%
The Toxicology Investigators Consortium Case Registry - The 2011 Experience	Wiegand TJ, et al. 2012 [57]	Portuguese man-of-war (jellyfish)				0
The Toxicology Investigators Consortium Case Registry - The 2012 Experience	Wiegand TJ, et al. 2013 [58]	Lionfish, Jellyfish (PMW)				0
2015 Annual Report of the American Association of Poison Control Center' National Poison Data System (NPDS): 33rd Annual Report	Mowry JB, et al. 2016 [60]	Fish stings		288, 46.5%	No effect (6, 0.97%) Minor (204, 33%) Moderate (110, 18%) Major (3, 0.5%)	1, 0.2%
		Cnidaria		72, 22%	No effect (4, 0.1%) Minor (120, 37%) Moderate (18, 0.6%) Major (0, 0%)	0
		Other/Unknown		51, 18.6%	No effect (41, 15%) Minor (29, 11%) Moderate (13, 5%) Major (1, 0.4%)	0
Mortality, hospital admission, and healthcare cost due to injury from venomous and non-venomous animal encounters in the USA: 5-year analysis of the National Emergency Department Sample	Forrester JD, et al. 2018 [61]	Venomous marine animals and plants		568, 2%	Injury severity score >15 11, 0.03%	0
Animal-Encounter Fatalities: United States, 1999-2016: Cause of Death and Misreporting	Haskell MG and Langley RL, 2020 [62]	Venomous marine animals and plants				UCD = 1, 0.03% MCD = 2, 0.05%
Envenomations during pregnancy reported to the national poison data system, 2009-2018	Ramirez-Cruz MP, et al. 2020 [64]	Fish stings Jellyfish/other coelenterates				0
2020 Annual Report of the National Poison Data System© (NPDS) from America's Poison Centers: 38th Annual Report.	Gummin DD, et al. 2021 [65]	Fish stings		240, 39%	No effect (8, 1.3%) Minor (214, 34.5%) Moderate (76, 12.3%) Major (2, 0.3%)	0
		Cnidaria		50, 19%	No effect (1, 0.4%) Minor (73, 28%) Moderate (22, 8.3%) Major (0, 0%)	0
		Other/Unknown		63, 11%	No effect (82, 14.8%) Minor (75, 13.5%) Moderate (20, 3.6%) Major (0, 0%)	0
2021 Annual Report of the National Poison Data System(©) (NPDS) from America's Poison Centers: 39th Annual Report.	Gummin DD, et al. 2022 [66]	Fish stings		214, 44%	No effect (17, 3.5%) Minor (167, 34.2%) Moderate (53, 10.9%) Major (2, 0.4%)	0
		Cnidaria		58, 22.4%	No effect (5, 1.9%) Minor (81, 31.3%) Moderate (23, 8.9%) Major (2, 0.78%)	0
		Other/Unknown		87, 17.4%	No effect (69, 13.8%) Minor (75, 15%) Moderate (20, 4%) Major (3, 0.6%)	0
Nationwide Aquatic Envenomations Reported to US Poison Control Centers from 2011 to 2020	Kirchberg TN, et al. 2024 [67]	Fish stings		Management site: On-site (4845, 57%) En route (2250, 26%) Referral to HCF (1140, 13%) Unknown (282, 3.3%) Level of care: Treated, released (2167, 64%) Lost to follow-up (718, 21%) Did not arrive at HCF (221, 7%) Noncritical care unit (218, 6%) Critical care unit (63, 2%) Psychiatric facility (3, 0.1%)	No effect (26, 0.5%) Minor (1770, 34.4%) Moderate (707, 14%) Major (9, 0.2%)	0
		Cnidaria			No effect (16, 0.6%) Minor (801, 32%) Moderate (254, 10%) Major (6, 0.2%)	0
		Other/Unknown			No effect (14, 2%) Minor (274, 33%) Moderate (82, 10%) Major (4, 0.5%)	0
South America						
Puncture wounds by driftwood catfish during bucket baths: local habits of riverside people and fish natural history in the Amazon	Sazima I, et al. 2005 [68]	Driftwood catfish		1 person needed to go to hospital to have broken spine surgically removed		N/A living sample
Injuries and envenomings by aquatic animals in fishermen of Coxim and Corumbá municipalities, State of Mato Grosso do Sul, Brazil: Identification of the causative agents, clinical aspects and first aid measures	Silva GC, et al. 2010 [47]	Catfish and Stingrays				N/A living sample
Trauma and envenoming caused by stingrays and other fish in a fishing community in Pontal do Paranapanema, State of Sao Paulo, Brazil: epidemiology, clinical aspects, and therapeutic and preventive measures	Haddad Junior V, et al. 2012 [71]	Stingrays		Treated at hospital or community health center 6, 100%	6 months for ulcers to heal	N/A living sample
Mortality caused by venomous animals in Venezuela: 1980-1999	De Sousa L, et al. 2014 [72]	Other - including venomous marine animals and plants				58 (3.9%) - does not indicate which species
Injuries caused by aquatic animals in Brazil: an analysis of the data present in the information system for notifiable diseases	Reckziegel GC, et al. 2015 [40]	Stingrays	<1 hours (1056, 37.2%) 1-3 (697, 24.5%) 3-6 (281, 9.9%) 6-12 (81, 2.9%) 12-24 (110, 3.9%) >24 (265, 9.3%) Not answered (352, 12.4%)			Not reported - accidents/injuries only
		Jellyfish/PMW	<1 hours (367, 68%) 1-3 (56, 10.4%) 3-6 (9, 1.7%) 6-12 (4, 0.7%) 12-24 (12, 2.2%) >24 (23, 4.3%) Not answered (69, 12.8%)			
		Toadfish	<1 hours (29, 15.9%) 1-3 (29, 15.9%) 3-6 (14, 7.7%) 6-12 (17, 9.3%) 12-24 (20, 11%) >24 (27, 14.8%) Not answered (45, 24.7%)			
		Catfish	<1 hours (25, 35.2%) 1-3 (7, 9.9%) 3-6 (5, 7%) 6-12 (2, 2.8%) 12-24 (4, 5.6%) >24 (18, 25.4%) Not answered (10, 14.1%)			
		Sea urchins	<1 hours (3, 17.6%) 1-3 (1, 5.9%) 3-6 (3, 17.6%) 6-12 (1, 5.9%) 12-24 (2, 11.8%) >24 (4, 23.5%) Not answered (3, 17.6%)			
		Other	<1 hours (154, 33%) 1-3 (97, 20.8%) 3-6 (39, 8.4%) 6-12 (20, 4.3%) 12-24 (42, 9%) >24 (61, 13.1%) Not answered (54, 11.6%)			
Injuries caused by the venomous catfish *pintado* and *cachara* (*Pseudopltaystoma* genus) in fishermen of the Pantanal region in Brazil	Aquino GN, et al. 2016 [73]	Catfish				N/A as living sample
Delayed healthcare and secondary infections following freshwater stingray injuries: risk factors for a poorly understood health issue in the Amazon	Sachett J, et al. 2018 [75]	Freshwater stingray	0-3 (322, 74.5%) 4-6 (55, 12.7%) 7-12 (8, 1.9%) 13-24 (5, 1.2%) >24 (42, 9.7%)	452, 100%		0
Epidemiology of aquatic animal poisonings reported to a Colombian toxicology control center	Montoya DV, et al. 2019 [76]	Stingrays and cnidaria				0
Injuries caused by fish to fishermen in the Vale do Alto Jurua, Western Brazilian Amazon	Costa TND, et al. 2020 [77]	Mandis (catfish) Stingray			Reported recovery range: 3 days (83, 40.7%) 2 weeks (104, 51%) 1-3 months (17, 8.3%) Catfish: Loss of mobility of hallux (foot) (1, 0.5%) Spines in palmar region of right hand (1, 0.5%)	N/A living sample
Mortality caused by venomous animals in Venezuela (2000-2009): A new epidemiological pattern	De Sousa L, et al. 2021 [78]	Other - including venomous marine animals and plants				84 (11.1%) - does not indicate which species
Temporal trend and epidemiological profile of accidents involving venomous animals in Brazil, 2007-2019	De Souza TC, et al. 2022 [79]	Other - including hymenoptera, beetles, centipedes, fish, and cnidaria				64 (0.07% of "Other" accidents, 0.003% TC) - does not indicate specific cause of death
Venomous animals in Pernambuco: children at risk	Albuquerque MCD, et al. 2022 [80]	Aquatic animals				1 (0.4%) - does not indicate which species
Europe						
Marine envenomations in returning French travelers seen in a tropical diseases unit, 2008-13.	Henn A, et al. 2016 [83]	Corals (11)	14 days between envenomation and consultation (Range 6-111)			N/A living sample
		Stonefish (10)	14.5 (Range 2-40)			
		Jellyfish (8)	14.5 (Range 7-130)			
		Miscellaneous (8), 2 stingray, 2 weever fish, 2 starfish, 1 sea anemone, 1 lionfish	13 (Range 10-24)			
Lifeguard assistance at Spanish Mediterranean beaches: Jellyfish prevail and proposals for improving risk management	Bordehore C, et al. 2016 [84]	Jellyfish Sea urchin Weever fish				Does not indicate cause of 7 deaths reported
Asia						
Poisoning in Israel: Annual Report of the Israel Poison Information Center, 2007	Bentur Y, et al. 2008 [86]	Fish Jellyfish Sea urchin				0
Venomous fish injuries along the Israeli Mediterranean coast: scope and characterization	Gweta S, et al. 2008 [87]	Stingray (24)		Hospitalized (28%) > 10 days (4)	Minor (29%) Moderate (53%) Major (9%) - including loss of a finger, permanent severe scarring, or inability to bend finger	N/A living sample
		Weever fish (17)				
		Rabbit fish (10)				
		Catfish (8)				
		Jellyfish (295) Fish (730) Unknown (163)	< 2h (55%) 2-8h (9.5%) 8-24h (16.4%) > 24h (12.1%)	Hospitalization for 10+ days (1, 4%)		Not reported
Environmental factors associated with the prevalence of animal bites or stings in patients admitted to an emergency department	Hsiao M-H, et al. 2012 [80]	Jellyfish				0
Poisoning in Israel: annual report of the Israel Poison Information Center, 2012	Bentur Y, et al. 2014 [88]	Fish Jellyfish Other				0
Poisoning in Israel: Annual Report of the Israel Poison Information Center, 2017	Bentur Y, et al. 2019 [90]	Fish Jellyfish Catfish Sea urchin Other/unknown				0
Africa						
South African Marine Envenomations and Poisonings as Managed Telephonically by the Tygerberg Poisons Information Centre: A 20-Year Retrospective Review	Marks CJ, et al. 2019 [91]	Bluebottle				1 (0.001%) Anaphylactic shock following bluebottle sting
Epidemiology of the cnidarian *Pelagia noctiluca* stings on Moroccan Mediterranean beaches	Mghili B, et al. 2020 [92]	Jellyfish		0		0
Tropical marine faunal hazard knowledge, incidents and associated health burden among seascape users at the Kenyan coastline	Kihia CM, et al. 2023 [93]	Lionfish			5.24 ± 1.53 (0-10)	Does not report
		Stonefish			8.52 ± 1.42 (0-10)	
		Stingray			6.72 ± 1.61 (0-10)	
		Urchin			4.21 ± 0.89 (0-10)	
		Jellyfish			4.37 ± 1.92 (0-10)	
		Sea snake			5.57 ± 1.92 (0-10)	
Oceania						
An analysis of marine animal injuries presenting to emergency departments in Victoria, Australia.	Taylor DM, et al. 2002 [37]	Jellyfish (42)		(1, 2%) Sting to the eye		0
		Stingrays (46)		(2, 4%)		
Leisure-related injuries at the beach: An analysis of lifeguard incident report forms in New Zealand, 2007-2012	Moran K and Webber J. 2014 [94]	Marine sting				Does not indicate what caused the deaths (17)
Injury trends from envenoming in Australia, 2000-2013	Welton RE, et al. 2017 [95]	Jellyfish				3, 5% of deaths by ENV, 0.008% of total ENV
Environmental Deaths in the Northern Territory of Australia, 2003-2018	Tiemensma M, 2019 [96]	Jellyfish (*Chironex fleckeri*)				1, 0.02% Total, 0.6% ENV
Animal bite wounds and their management in tropical Australia	Vardanega J, et al. 2022 [97]	Jellyfish (129)		Hospitalized (129, 100%) ICU admission (5, 3.9%) Unplanned readmission (2, 2%)	Significant trauma (0, 0%)	0
		Stonefish (22)	Presentation > 8h (1, 5%) Presentation > 24h (4, 18%)	Hospitalized (22, 100%) Unplanned readmission (2, 9%)	Significant trauma (1, 5%)	
		Stingray (17)	> 8h (7, 41%) > 24h (8, 47%)	Hospitalized (17, 100%) Unplanned readmission (1, 6%)	Significant trauma (3, 18%)	
Australian Sea Snake Envenoming Causes Myotoxicity and Non-Specific Systemic Symptoms-Australian Snakebite Project (ASP-24)	Johnston C, et al 2022 [98]	Sea snake (13)	Elapsed time to AV (hours) 1-5 (4, 30.8%) 6-12 (3, 23.1%) > 12 (1, 7.7%)	Time to discharge post-bite (hours) 0-12 (2, 15.4%) 13-24 (6, 46.2%) 25-36 (3, 23.1%) 37-48 (1, 7.7%) 48+ (1, 7.7%)		0

Morbidity and mortality information of those that experienced a bite or sting. HCF: health care facility; UCD: underlying cause of death; MCD: multiple causes of death; ENV: envenomation.

Morbidity and injury severity was documented in thirteen studies (Table 5) [51, 54, 55, 60, 61, 65-67, 71, 77, 87, 93, 97]. In reports to poison control centers in Texas, as well as across the USA, aquatic envenomations by stingrays [51], fish and coelenterates (jellyfish, corals, anemones) [54, 55, 60, 65-67] resulted in mostly minor effects. Significant trauma ranged from 0.03% to 18% depending on the envenomating species and population [61, 97]. Major morbidity was reported by 9% of fisherfolk along the Israeli Mediterranean coast, including loss of a finger, permanent severe scarring and the restricted mobility of digits [87]. Catfish injury also caused loss of mobility in the foot for one fisherman in Brazil [77]. Two studies investigated days of work lost or financial cost associated with stings or bites [47, 73]. In the Corumbá and Miranda municipalities of Brazil, 41% and 76% of fishermen continued to work while injured, respectively [73]. Fewer fishermen missed up to one week of work (11% in Miranda, 41% in Corumbá) [73]. Similarly, 44% of fishermen from the Coxim and Corumbá missed less than a week of work due to envenomings [47]. Only 6% and 12% of fishermen missed 7-15 days or 15-29 days, respectively [47].

Mortality data was included in thirty-three articles, the majority of which reported zero deaths due to venomous aquatic animals (Table 5) [41, 50, 51, 54-58, 60, 61, 62, 64-67, 72, 75, 76, 78-80, 84, 86, 88-92, 94-98]. Fatalities were reported in thirteen articles [50, 56, 60, 62, 72, 78-80, 84, 91, 94-96]. However, the description of the responsible organism(s) varied or was not included. Three articles categorized offending animals as “venomous marine animals and plants” and two articles further grouped these with other categories of unknown or unspecified venomous animals [50, 56, 62, 72, 78]. These groups were responsible for a total of 146 deaths [50, 56, 62, 72, 78]. A total of four fatalities due to box jellyfish were reported from two articles [95, 96], while another article reported one death following a blue-bottle (*Physalia*) sting [91]. Overall, the fatalities reported accounted for 0.001% to 11.1% of total deaths, depending on the population investigated, with the lowest percent reported from total telephone consultations made to the Tygerberg (South Africa) Poisons Information Center [91], and the highest reported in Venezuela, evaluating mortality caused by venomous animals between 2000-2009 [78]. Ten articles were studies of living human subjects, and thus mortality was not assessed. Six articles did not report mortality, and three articles specifically excluded this information.

## Discussion

To document the health burden of aquatic envenomation in terms of incidence and prevalence of envenomation, as well as high-risk geographic regions and populations, fifty-three articles were analyzed. Several studies highlighted the need for more research, as well as higher quality data, to establish envenomating burden [53, 80, 84, 87, 94]. This review revealed important limitations in the existing research, with the counter-productive focus on low to zero risk, economically-advantaged populations and a paucity of data from high-risk populations. Given the currently available literature, this review can identify important research gaps and future research directions.

The 53 eligible publications represent studies of aquatic envenomations primarily from the Americas (33), in the United States and Brazil, as well as Europe (5), Asia (6), Africa (3), and Oceania (6). Populations were limited to commercial fishermen [52], artisanal or small-scale fisherfolk [47, 68, 71, 73, 77, 87], beachgoers [82, 84, 85, 92-94], and general populations attending emergency departments or hospitals [41, 53, 59, 61, 63, 81, 88, 95, 97, 98], or calling poison control centers [51, 54, 55, 57, 58, 60, 65, 66, 67, 70, 76, 80, 86, 87, 89-91]. While these data constitute direct measurements of defined populations, they do not include extensive research from lower- and middle-income countries, especially in critical geographic tropical regions such as Indonesia, Malaysia, and the Philippines. For these reasons, this systematic review allows for few comparisons across populations groups and geographic regions, limiting the general characterization of aquatic envenomation injury burden.

A number of studies did examine envenomations among fisherfolk and those treated in EDs. Fisherfolk are an occupational group clearly at risk of aquatic envenomation in both freshwater [40, 74, 75] and marine environments [47, 52, 87]. The prevalence of envenomations among fisherfolk described in this review typically exceeded 70% (Figure 3B) [47, 52, 71, 73, 77, 87]. However, research among fisherfolk was dominated by a single group in Brazil primarily working with riverine populations. While their research tended to include entire communities, sample sizes were relatively small (from n = 39 to 481) [47, 71, 73]. Across the studies, the fisherfolk sampled were artisanal or small-scale. Interestingly, five studies reported repeated stings among fisherfolk, highlighting the importance of following high-risk groups over time [52, 68, 71, 73, 77]. When included, the sex of those surveyed was almost exclusively male (74-100%) [52, 74, 75, 77]. Despite the fact that women also fish, often process them [99, 100] and are disproportionately represented in gleaning activities [101], research on the topic is overlooked. Aquatic envenomations are clearly a concern for fisherfolk and possibly, a serious source of recurrent injury. However, studies with larger sample sizes are needed, in more diverse locations and of different types of fisherfolk, such as those working for commercial fleets, as well as a focus on women specifically. Further, longitudinal studies are necessary to calculate incidence, and whether rates of envenomation events are changing over time.

In contrast to fisherfolk surveys, few aquatic envenomation events were recorded in samples from EDs. Typically, these studies had large sample sizes and most (71%) reviewed several years of data [41, 53, 59, 61, 63]. The denominator for these studies represented the general population of those with access to medical care for urgent health needs in a given location and with limited risk of envenomation, sharply contrasting that of fisherfolk. Such samples include people with no exposure to aquatic envenomating organisms, such as those who do not go to the beach. Based on these studies, which were conducted in high income settings (e.g., US, Italy, and Taiwan), aquatic envenomations are not a significant source of morbidity in emergency medical settings [41, 53, 59, 61, 63, 81, 88].

Lifeguard reports or surveys of beachgoers may provide a more accurate representation of the burden of injury from aquatic envenomation and demonstrate the importance of appropriate population selection. The numerous life-guard reports summarized in articles from Spain, Morocco, and New Zealand demonstrate that envenomation can be an important source of injury (16-70%) at the beach [84, 85, 92, 94]. However, lifeguard reports likely still underestimate burden, as many victims may not report to lifeguard stations, or they may occur at beaches that do not have these services [84, 85]. These studies provide evidence that envenomation events do not translate into ED visits, such as when the injury is minor or when victims choose not to, cannot afford, or are too far from medical facilities. An article examining fisherfolk and farmers in the Tapajós River Basin of the Amazon reported that 68% of fisherfolk stung did not seek healthcare; the reasons were not specified [74]. In two US articles, the findings showed that those with private or self-pay health insurance more commonly experienced envenomation [52, 61], which could mean these groups were at higher-risk, or that they were more likely to go and/or have the financial means to seek care following an incident.

There is a necessity for encompassing systems of reporting to capture all populations and determine burden. Brazil’s SINAN is an example of a comprehensive surveillance system used to track disease and injury [102, 103]. Three articles in this review used SINAN data, reporting that 88.7% of aquatic injuries were due to venomous animals [40], and that throughout the study period there was a rising temporal trend of accidents involving venomous animals [79]. The third article focused on stingray envenomation specifically, reporting between 20 to 77 cases of envenomation per 100,000 inhabitants in three communities in the Amazon [75]. An important aspect of SINAN data is that it contains sociodemographic information. All three of the SINAN-based articles documented higher frequencies of envenomation among younger populations (from < 5 to 34) and men [40, 75, 79]. Sachett *et al.* [75] also described risk-factors for secondary infection following stingray envenomation including occupation, ethnicity and time to treatment [75]. Work-related injuries and increased time to treatment, but not ethnicity, were significantly associated with increased risk of secondary infections [75]. Out of those envenomed, 57% worked in maintenance and repair services, while 39% were farmers and fishermen [75]. This type of research provides essential information on who is at risk of envenomation and serious sequelae. Aside from fisherfolk and beachgoers, there were no studies focused on presumed higher-risk groups including those whose jobs require work in aquatic environments, such as marine tour operators (e.g. scuba and snorkel instructors), marine and aquatic biologists, certain types of military personnel, and underwater ship repair personnel. In all cases, research on envenomation would benefit by including more details on the characteristics of victims as compared to non-victims in order to clarify at-risk populations, which is needed to tailor prevention and treatment efforts.

In the case of envenomation specifically and injuries generally, disability is a function of the timing and quality of treatment, secondary to the severity of the injury, and the general susceptibility of the individual (underlying health, age, etc.) [104]. While this review included six articles describing the elapsed time between envenomation and treatment, as well as fifteen describing common treatments, the only correlation with subsequent disability or morbidity was looking at secondary infection following stingray envenomation [75]. This review showed widely varying first-aid tactics: fish parts, smoke, gasoline, and ice (Table 4) [47, 71, 73, 77, 82, 92]. Importantly, numerous articles listed hot-water or heat as a treatment [47, 71, 73, 74, 77, 82, 87], an approach supported by the literature for treatment of cnidaria (sea anemones, corals, jellyfish, hydroids), weever fish, stingrays, scorpionfish and general marine stings due the thermolabile nature of these venoms [105-109]. For all aquatic envenomations, the first step in management is removing the victim from the water and administering any necessary life support [109, 110]. For jellyfish stings, removal of the tentacles by vinegar or copper-gluconate containing spray [110] should be performed once an initial evaluation is completed, followed by application of copper-gluconate containing cream [110] or hot-water treatment for at least 45 minutes [105-108, 110-115]. There are numerous case reports of aquatic envenomations, commonly presented with treatments and outcomes [116-120], but a scarcity of literature that also includes the frequencies of envenomation. Many articles on marine injuries provided epidemiological details on those injured, but did not provide denominator data to determine frequency [11, 51, 121-129]. It is impossible to draw reasonable conclusions about the burden of injury from envenomations without these details.

While immediate and optimal first aid treatment is critical in minimizing victim injury and improving outcomes, education and prevention methods can also reduce envenomation events. Australia is an example of a high-risk region that utilizes public health efforts to minimize aquatic envenomations. Informed by knowledge of animal ecology, including seasonality, between November to May, marine stinger enclosures made up of 25 mm mesh are put out to exclude box jellyfish from the area [130, 131]. Further, signs are posted at high-risk beaches, and donning Lycra-suits prior to swimming is recommended to the public [130, 131]. Similarly, jellyfish flags are displayed on beaches in Spain, and there is ongoing communication between academics and beach authorities to guide management [85]. These prevention efforts may provide a useful example for other high-risk regions.

There was an important gap in the geographic distribution of studies on this topic. While venomous fauna are most abundant in tropical waters [46], such as in the coral triangle [132], Oceania, and the Caribbean, the research was limited to a single study in the Philippines and six within Oceania. There was a complete lack of research from Micronesia, Polynesia, and Melanesia, yet, the highest global mortality estimate due to venomous animal contact, as reported by the 2019 Global Burden of Disease Study, was located in Palau at the northeastern margin of the coral triangle (4.95 deaths/100,000 people) [25, 133]. Islanders as well as Indigenous Peoples more generally, are likely to experience higher burdens of envenomation incidents. This is supported by envenomation research inclusive of coastal Indigenous populations in Mtwapa and Gazi, Kenya, who face many barriers to care including low health coverage, limited access to potable water, doctor shortages (1 doctor to 600 patients), and long distances to health facilities [93]. Further, stingray envenomation in riverine communities of South America are more likely to go unreported, due to the low mortality and the remote location of the incidents [44]. Further, these populations may go to traditional healers and therefore not be captured by formal reporting [69]. Thus, Islander and Indigenous populations are important populations to include in future studies of marine envenomation.

In Oceania, there were five articles from Australia and a single article from New Zealand. A total of four deaths since 2000 were reported from two Australian articles [95, 96]. However, during this same time period, news stories and case reports totaled at least 14 deaths [1-7]. Further, in regions of the coral triangle including Thailand, the Philippines, and Malaysia, there were 20-30 deaths described by social media outlets and case reports that were not included in our review [8-24]. Unfortunately, these incidents are not quantified, for example, in terms of the general population or beachgoers, and therefore did not meet our inclusion criteria. While these deaths are occurring, they were not captured by formal reporting or included in published, peer-reviewed literature. The one, small study using a convenience sample in the Philippines indicated that serious envenomations may be common in certain coastal communities [36]. Thus, in the areas of the world presumed to have some of the highest risks of serious marine envenomation there is almost no research on the burden of injury.

The work that is available on this topic is predominantly for jellyfish including Portuguese man-of-war or blue bottle, *Pelagia noctiluca, Carybdea marsupialis*, and box jellyfish [36, 41, 55, 57, 58, 60, 65, 66, 67, 69, 70, 76, 79, 82, 83, 85, 91, 92, 95, 96], stingrays [40, 41, 47, 51-53, 59, 63, 71, 74, 75-77, 87, 91, 93, 97] and catfish [40, 47, 52, 63, 68, 71, 73, 77, 87, 90, 93]. Other venomous marine animals are understudied: cone snails, blue-ringed octopus, sea snakes, stone fish, lionfish, etc. This is concerning for multiple reasons. First, certain occupational groups, as we have seen reported amongst fisherfolk, have elevated exposure to these organisms. Second, marine tourism including swimming, snorkeling, and diving is increasing globally [134, 135]. Third, 40% of the global population resides within 100 km of the coast and this percentage is increasing [43]. Additionally, climate change is predicted to lead to increased populations of venomous marine species, therefore increasing the likelihood of encounters [136]. The overall burden of aquatic envenomation, as well as the contribution from individual species, is necessary to determine effective preventative measures and treatment efforts.

## Limitations

Our review only included articles in English, French, Spanish, and Portuguese, using English search terms, limiting the inclusion of potential published literature from the coral triangle. These regions may have additional articles in Thai, Tagalog, Indonesian, or Malay that were not included. Database selection was limited by institutional access, thus preventing a search using Scopus. However, given that the four databases searched yielded 6237 unique articles, it is unlikely that the exclusion of Scopus significantly altered the analysis or conclusions. Only published scientific manuscripts were reviewed and thus this review did not include data on aquatic envenomation events that might be available in other sources, such as hospital records, government surveillance and reports that were not peer-reviewed. For example, in high-income settings, electronic medical records could be queried to quantify envenomations treated in emergency rooms (ICD-9 codes V5889 and V9895; ICD-10 codes W53-W59 and X20-X29) and then be compared to the overall population seeking emergency care. While this is certainly a suggestion for future research, it does not address the likely much higher burden of envenomation injuries in communities with limited access to quality healthcare. Further, even if additional unpublished data are available in some settings, the lack of scientific productivity on the topic indicates a lack of prioritization for it, in terms of funding, research activity, and publication. Thus, this review highlights an important topic in injury research that is largely ignored by the scientific community.

## Conclusion

Research on aquatic envenomation burden, describing specific populations and geographic hot spots, is limited. This systematic review is the first to analyze and summarize the nearly 25 years of extant literature to answer these questions. Overall, it demonstrates that certain population groups may be at high risk of envenomation (e.g., fisherfolk and beachgoers generally). However, there is a predominance of research examining general populations, which includes groups at low to no risk of aquatic envenomation. There is a lack of research in geographic areas believed to have high envenomation risks. Further, there is insufficient research on sociodemographic characteristics that may be associated with envenomation risk. In the absence of better data on envenomation risk and burden, it is difficult to make evidence-informed decisions to direct resources and develop programs to prevent envenomation by aquatic species, as well as improve first-aid and treatment when envenomation occurs.

### Abbreviations

ED: emergency department; NTD: neglected tropical disease.

## Data Availability

All data generated or analyzed during this study are included in this article.

## Supplementary material

The following online material is available for this article:

Additional file 1 Search terms used in this systematic review.

Additional file 2 - Aquatic envenomation frequency as a percentage of the total poison control calls.

Additional file 3 - Aquatic envenomation frequency as a percentage of the total envenomations reported to poison control centers.

Additional file 4 - Sting locations.

Additional file 5 - Symptoms of envenomation.
