# Supplementary material for: A systematic review of reports on aquatic envenomation: are there global hot spots and vulnerable populations?
Source: J Venom Anim Toxins Incl Trop Dis. 2024 Dec 20;30:e20240032. doi: 10.1590/1678-9199-JVATITD-2024-0032 (PMC11730067; doi:10.1590/1678-9199-JVATITD-2024-0032)
Supplement: Additional file 1 [file 1678-9199-jvatitd-30-e20240032-s1.pdf]

J Venom Anim Toxins incl Trop Dis
https://doi.org/10.1590/1678-9199-JVATITD-2024-0032

Supplementary Material to "A systematic review of reports on aquatic envenomation: are there global hot spots and vulnerable populations?"

Additional file 1. Search terms used in this systematic review.

("Scorpion Stings"[Mesh] OR "Snake Bites"[Mesh] OR "Spider Bites"[Mesh])

OR

((jellyfish OR medusozoa OR medusozoan OR "sea jellies" OR cnidaria OR cnidarians OR "sea anemone" OR "sea anemones" OR anemone OR anemones OR "sea urchin" OR "sea urchins" OR cubozoa OR cubozoans OR snake OR reptile OR platypus OR lizard OR frog OR amphibian OR spider OR scorpion OR arachnid OR octopus OR octopuses OR cephalopod OR cephalopods OR stingray OR stingrays OR myliobatoidei OR stonefish OR lionfish OR "sea snake" OR "sea krait" OR hydrophiinae OR scorpaeniformes OR scorpionfish OR scorpaenidae OR cone[tiab] OR "cone snail" OR conus OR catfish OR catfishes OR siluriformes OR sea cucumbers OR echinoderm OR "crown of thorns" OR starfish OR shrew OR mole OR weever fish OR rabbitfish OR rabbitfishes OR siganidae OR dogfish OR squalidae OR toadfish OR "Speleonectes tulumensis" OR crustacean)

AND

(toxin OR toxins OR envenomation OR envenomations OR stings OR sting OR venom OR venoms OR poison))

AND

(mortality OR mortalities OR morbidity OR morbidities OR burden OR burdens OR prevalence OR prevalences OR epidemiology OR epidemiologies OR incidence OR incidences)
