# Supplementary material for: A systematic review of reports on aquatic envenomation: are there global hot spots and vulnerable populations?
Source: J Venom Anim Toxins Incl Trop Dis. 2024 Dec 20;30:e20240032. doi: 10.1590/1678-9199-JVATITD-2024-0032 (PMC11730067; doi:10.1590/1678-9199-JVATITD-2024-0032)
Supplement: Additional file 2 - [file 1678-9199-jvatitd-30-e20240032-s2.pdf]

J Venom Anim Toxins incl Trop Dis
https://doi.org/10.1590/1678-9199-JVATITD-2024-0032

Supplementary Material to "A systematic review of reports on aquatic envenomation: are there global hot spots and vulnerable populations?"

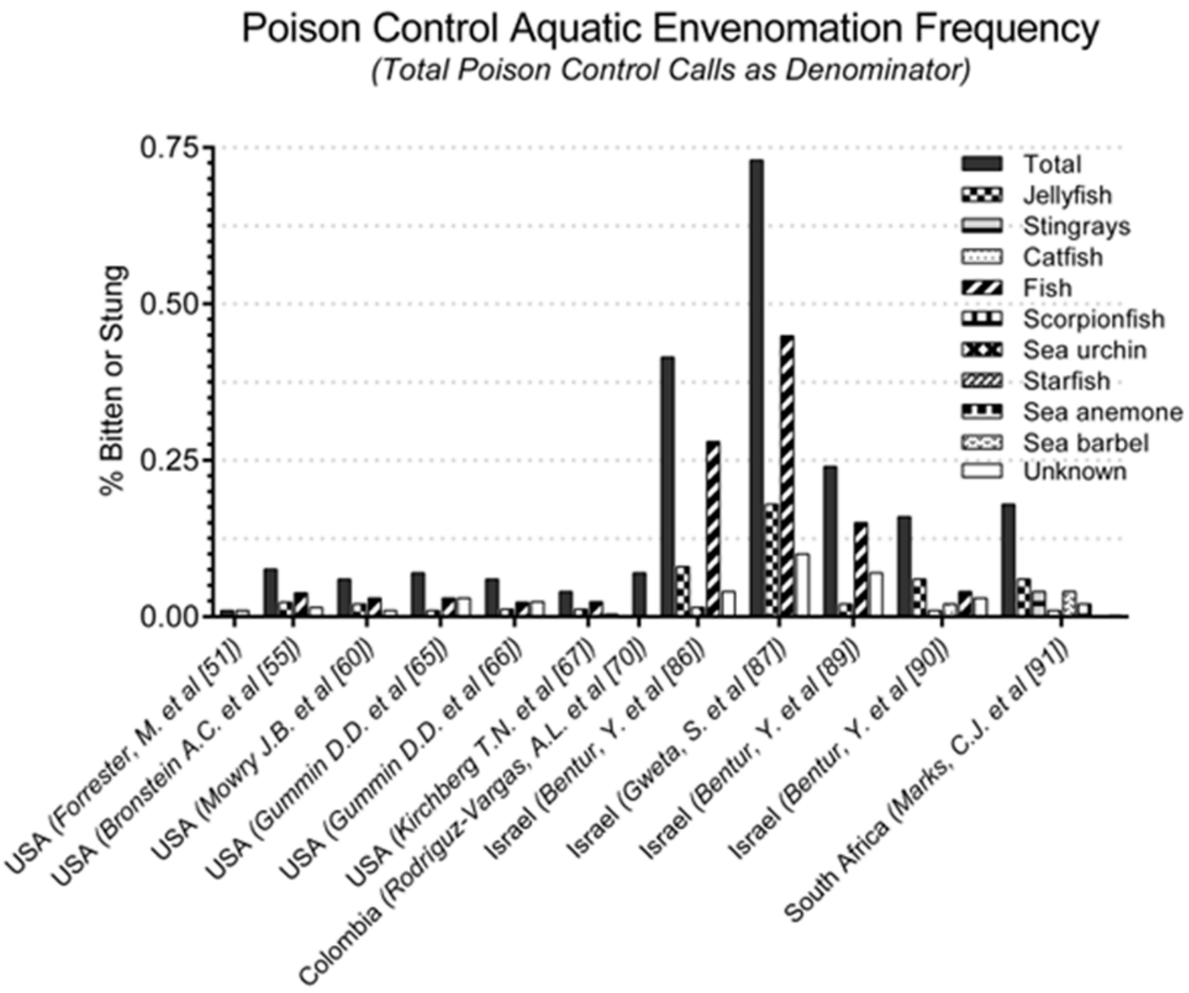

Additional file 2. Aquatic envenomation frequency as a percentage of the total poison control calls.
