# Supplementary material for: A systematic review of reports on aquatic envenomation: are there global hot spots and vulnerable populations?
Source: J Venom Anim Toxins Incl Trop Dis. 2024 Dec 20;30:e20240032. doi: 10.1590/1678-9199-JVATITD-2024-0032 (PMC11730067; doi:10.1590/1678-9199-JVATITD-2024-0032)
Supplement: Additional file 4 - [file 1678-9199-jvatitd-30-e20240032-s4.pdf]

J Venom Anim Toxins incl Trop Dis

https://doi.org/10.1590/1678-9199-JVATITD-2024-0032

Supplementary Material to "A systematic review of reports on aquatic envenomation: are there global hot spots and vulnerable populations?"

Additional file 4. Sting locations.

Title	Author, year published [ref.]	Organism(s)	Hand and arm bitten or stung	Foot and leg bitten or stung	Torso bitten or stung	Other body part bitten or stung
**North America**						
Skin problems related to the occupation of commercial fishing in North Carolina	Burke WA et al., 2006 [52]	Jellyfish (22)				Eyes (21, 95%)<br>Face/chest (1, 5%)
Mortality, hospital admission, and healthcare cost due to injury from venomous and non-venomous animal encounters in the USA: 5-year analysis of the National Emergency Department Sample	Forrester JD et al., 2018 [61]	Venomous marine animals and plants	Isolated extremity (376, 1%)		Isolated head/neck (17, 0.05%)	Isolated external (24, 0.07%)<br>Multiple body regions<br>(34454, 99%)
**South America**						
Puncture wounds by driftwood catfish during bucket baths: local habits of riverside people and fish natural history in the Amazon	Sazima I et al., 2005 [68]	Driftwood catfish	Upper limb (12, 52%)	Lower limb (4, 17%)	Torso (3, 13%)	Head and Neck (4, 17%)

Title	Author, year published [ref.]	Organism(s)	Hand and arm bitten or stung	Foot and leg bitten or stung	Torso bitten or stung	Other body part bitten or stung
Trauma and envenoming caused by stingrays and other fish in a fishing community in Pontal do Paranapanema, State of Sao Paulo, Brazil: epidemiology, clinical aspects, and therapeutic and preventive measures	Haddad Junior V et al., 2012 [71]	Stingrays		> 3		
Injuries caused by aquatic animals in Brazil: an analysis of the data present in the information system for notifiable diseases	Reckziegel GC et al., 2015 [40]	Total	Hand (407, 10%)<br>Forearm (53, 1%)	Foot (2785, 68%)<br>Leg (368, 9%)	(130, 3%)	Head/thigh/arm (231, 6%)<br>Not specified (144, 4%)
		Stingrays	Hand (82, 3%)<br>Forearm (14, 1%)	Foot (2375, 84%)<br>Leg (231, 8.1%)	(6, 0.2%)	Head/thigh/arm (55, 2%)<br>Not specified (79, 3%)
		Jellyfish/PMW	Hand (70, 13%)<br>Forearm (30, 6%)	Foot (37, 7%)<br>Leg (109, 20%)	(116, 22%)	Head/thigh/arm(153, 28%)<br>Not specified (25, 5%)
		Toadfish	Hand (82, 45%)<br>Forearm (1, 1%)	Foot (72, 40%)<br>Leg (3, 2%)	(1, 1%)	Head/thigh/arm (3, 2%)<br>Not specified (19, 10%)
		Catfish	Hand (28, 39%)<br>Forearm (2, 3%)	Foot (29, 41%)<br>Leg (2, 3%)	(0, 0%)	Head/thigh/arm) (5, 7%)<br>Not specified (5, 7%)
		Sea urchins	Hand (2, 12%)<br>Forearm (1, 6%)	Foot (9, 53%)	(1, 6%)	Head/thigh/arm) (2, 12%)<br>Not specified (2, 12%)
		Other	Hand (143, 31%)<br>Forearm (5, 1%)	Foot (263, 56%)<br>Leg (23, 5%)	(6, 1%)	Head/thigh/arm) (13, 3%)<br>Not specified (14, 3%)
Injuries caused by the venomous catfish pintado and cachara (Pseudopltaystoma genus) in fishermen of the Pantanal region in Brazil	Aquino GN et al., 2016 [73]	Catfish	(58, 39%)	(53, 36%)		
Delayed healthcare and secondary infections following freshwater stingray injuries: risk	Sachett J et al., 2018 [75]	Freshwater stingray		"Most patient injuries occurred on the lower leg or foot" (Discussion)		

Title	Author, year published [ref.]	Organism(s)	Hand and arm bitten or stung	Foot and leg bitten or stung	Torso bitten or stung	Other body part bitten or stung
factors for a poorly understood health issue in the Amazon						
Epidemiology of aquatic animal poisonings reported to a Colombian toxicology control center	Montoya DV et al., 2019 [76]	Cnidaria		(1, 100%)		
		Stingray	(2, 18%)	(9, 82%)		
Injuries caused by fish to fishermen in the Vale do Alto Jurua, Western Brazilian Amazon	Costa TND et al., 2020 [77]	Mandis (catfish) Stingray	Arm (3.4%) – Catfish, piranha, bicos de pato, lustrosas and douradas	Feet (33.3%) – Stingray, catfish, piranha, electric fish Thigh (1%) – Catfish	Abdomen (0.5%) – catfish	0.5% of mandi-related to the gluteal region
**Europe**						
Impact of stinging jellyfish proliferations along South Italian Coasts: human health hazards, treatment and social costs	De Donno A et al., 2014 [82]	Jellyfish (1733)	Upper limb (398, 23%)	Lower limbs (693, 41%)	Abdomen (34, 2%)<br>Thorax (69, 3%)<br>Back (17, 1%)	Face (294, 17%)<br>Multiple (225, 13%)
Marine envenomations in returning French travellers seen in a tropical diseases unit, 2008-13	Henn A et al., 2016 [83]	Stonefish (10)		(10, 100%)		
**Asia**						
Venomous fish injuries along the Israeli Mediterranean coast: scope and characterization	Gweta S et al., 2008 [87]	Stingray (24)	Most common site of injury for all fish species – but no numerical value provided	Legs (9, 38%)	(1, 4%)	
		Weever fish (17)		Legs (2, 12%)		
		Rabbit fish (10)		Legs (1, 10%)		
		Catfish (8)				

Title	Author, year published [ref.]	Organism(s)	Hand and arm bitten or stung	Foot and leg bitten or stung	Torso bitten or stung	Other body part bitten or stung
**Africa**						
Epidemiology of the cnidarian Pelagia noctiluca stings on Moroccan Mediterranean beaches	Mghili B et al., 2020 [92]	Jellyfish	Upper limbs (357, 27%)	Lower limbs (700, 53%)	Thorax (40, 3%)<br>Abdomen (26, 2%)<br>Back (106, 8%)	Face (26, 2%)<br>Multiple sites (66, 5%)
**Oceania**						
An analysis of marine animal injuries presenting to emergency departments in Victoria, Australia	Taylor DM et al., 2002 [41]	Jellyfish (42)	Hand (2, 5%)<br>Arm (12, 29%)	Foot (5, 12%)<br>Leg (9, 21%)	(4, 10%)	Head (2, 5%)<br>Multiple locations (6, 14%)
		Stingrays (46)	Hand (10, 22%)<br>Arm (3, 7%)	Foot (18, 39%)<br>Leg (12, 26%)		Missing/unspecified (3, 7%)
		Sea urchin (7)	Hand (1, 14%)	Foot (6, 86%)		
		Coral (3)	Hand (1, 33%)<br>Arm (1, 33%)			Missing/unspecified (1, 33%)
		Fish (83)	Hand (55, 66%)<br>Arm (2, 2%)	Foot (15, 18%)<br>Leg (5, 6%)		Missing/unspecified (6, 7%)
Leisure-related injuries at the beach: An analysis of lifeguard incident report forms in New Zealand, 2007-2012	Moran K and Webber J, 2014 [94]	Marine sting	Extremities (hands & feet) (894, 65%)			
Animal bite wounds and their management in tropical Australia	Vardanega J et al., 2022 [97]	Jellyfish (129)	Arm (28, 22%)			
		Stonefish (22)		Foot/ankle (14, 63%)		
		Stingray (17)		Foot/ankle (8, 47%)		
		Fish (35)	Hand/wrist (22, 63%)			
Australian Sea Snake Envenoming Causes Myotoxicity and Non-Specific	Johnston C et al., 2022 [98]	Sea snake	Hand/finger (9, 69%)	Ankle/foot/leg (3, 23%)		Face (1, 7.7%)

Title	Author, year published [ref.]	Organism(s)	Hand and arm bitten or stung	Foot and leg bitten or stung	Torso bitten or stung	Other body part bitten or stung
Systemic Symptoms-Australian Snakebite Project (ASP-24)						
