# Supplementary material for: A systematic review of reports on aquatic envenomation: are there global hot spots and vulnerable populations?
Source: J Venom Anim Toxins Incl Trop Dis. 2024 Dec 20;30:e20240032. doi: 10.1590/1678-9199-JVATITD-2024-0032 (PMC11730067; doi:10.1590/1678-9199-JVATITD-2024-0032)
Supplement: Additional file 5 - [file 1678-9199-jvatitd-30-e20240032-s5.pdf]

https://doi.org/10.1590/1678-9199-JVATITD-2024-0032

Supplementary Material to "A systematic review of reports on aquatic envenomation: are there global hot spots and vulnerable populations?"

Additional file 5. Symptoms of envenomation.

Title	Author, year published [ref.]	Organism(s)	Pain	Edema	Erythema	Fever	Pruritus	Malaise	Shock	Nausea and vomiting	Neuro symptoms	Muscular spasms	Non-specific systemic symptoms	Necrosis	Respiratory failure and pulmonary edema	Blurred vision/dizziness	Conjunctivitis	Wound soiling	Ulcers, lesions, or bleeding	Secondary infection
**North America**																				
Skin problems related to the occupation of commercial fishing in North Carolina	Burke WA, et al. 2006 [52]	Jellyfish only	Pain/edema causing temporary ocular closure (21, 95%)	Pain/edema causing temporary ocular closure (21, 95%)					(1, 5%)							Temporary blindness, ocular closure (21, 95%)	(21, 95%)			
**South America**																				

Title	Author, year published [ref.]	Organism(s)	Pain	Edema	Erythema	Fever	Pruritus	Malaise	Shock	Nausea and vomiting	Neuro symptoms	Muscular spasms	Non-specific systemic symptoms	Necrosis	Respiratory failure and pulmonary edema	Blurred vision/dizziness	Conjunctivitis	Wound soiling	Ulcers, lesions, or bleeding	Secondary infection
Puncture wounds by driftwood catfish during bucket baths: local habits of riverside people and fish natural	Sazima I, et al. 2005 [68]	Driftwood catfish	Yes, but no numerical value	(3, 18%)																(3, 18%)
Trauma and envenoming caused by stingrays and other fish in a fishing community in Pontal do Paranapanema, State of Sao Paulo, Brazil: epidemiology, clinical aspects, and therapeutic and preventive measures	Haddad Junior V, et al. 2012 [71]	Stingrays	(6, 100%)																(6, 100%)	

Title	Author, year published [ref.]	Organism(s)	Pain	Edema	Erythema	Fever	Pruritus	Malaise	Shock	Nausea and vomiting	Neuro symptoms	Muscular spasms	Non-specific systemic symptoms	Necrosis	Respiratory failure and pulmonary edema	Blurred vision/dizziness	Conjunctivitis	Wound soiling	Ulcers, lesions, or bleeding	Secondary infection
Injuries caused by the venomous catfish *pintado* and *cachara* (*Pseudoplatystoma* genus) in fishermen of the Pantanal region in Brazil	Aquino GN, et al. 2016 [73]	Catfish	Radiating pain to the limb Corumbá (83, 75%) Miranda (23, 60%)	Corumbá (96, 87%) Miranda (31, 82%)	Corumbá (108, 98%) Miranda (31, 84%)	Corumbá (39, 35%) Miranda (30, 78%)	Corumbá (1, 1%) Miranda (19, 50%)	Corumbá (20, 18%) Miranda (2, 5%)	Cold sweat - Corumbá (72, 65%) Miranda (22, 57%) Tachycardia - Corumbá (54, 49%) Miranda (13, 34%)	Corumbá (2, 2%) Miranda (2, 5%)	Paresthesia - Corumbá (84, 76%) Miranda (16, 42%)			Corumbá (28, 26%) Miranda (9, 26%)	Yes; Dyspnea - Corumbá (8, 7%) Miranda (2, 5%)					Corumbá (6, 5%) Miranda (6, 16%)
Injuries caused by freshwater stingrays in the Tapajos River Basin: a clinical and sociodemographic study	Abati PAM, et al. 2017 [74]	Freshwater stingray	Intense pain (18, 95%)	(8, 42%)	(8, 42%)														(12, 62%)	

Title	Author, year published [ref.]	Organism(s)	Pain	Edema	Erythema	Fever	Pruritus	Malaise	Shock	Nausea and vomiting	Neuro symptoms	Muscular spasms	Non-specific systemic symptoms	Necrosis	Respiratory failure and pulmonary edema	Blurred vision/dizziness	Conjunctivitis	Wound soiling	Ulcers, lesions, or bleeding	Secondary infection
Delayed healthcare and secondary infections following freshwater stingray injuries: risk factors for a poorly understood health issue in the Amazon	Sachett J, et al. 2018 [75]	Freshwater stingray	(445, 99%)	(293, 65%)	Hyperemia (14, 3%)	(1, 0.2%)			Siderosis (1, 0.2%)	(3, 1%)				(17, 4%)		(8, 4%)			Bleeding (57, 12%)<br>Ecchymosis (70, 16%)	(40, 9%)
Epidemiology of aquatic animal poisonings reported to a Colombian toxicology control center	Montoya DV, et al. 2019 [76]	Stingray	(11, 100%)	(6, 55%)	(4, 36%)		(1, 9%)												Bleeding (1, 9%)	Dermonecrosis (1, 9%)<br>Cellulitis (1, 9%)<br>Other infection (2, 18%)
		Cnidaria	(1, 100%)		(1, 100%)	(1, 100%)														

Title	Author, year published [ref.]	Organism(s)	Pain	Edema	Erythema	Fever	Pruritus	Malaise	Shock	Nausea and vomiting	Neuro symptoms	Muscular spasms	Non-specific systemic symptoms	Necrosis	Respiratory failure and pulmonary edema	Blurred vision/dizziness	Conjunctivitis	Wound soiling	Ulcers, lesions, or bleeding	Secondary infection
Injuries caused by fish to fishermen in the Vale do Alto Jurua, Western Brazilian Amazon	Costa TND, et al. 2020 [77]	Catfish & Stingray	(204, 100%)		Hemorrhage (204, 100%)															
**Europe**																				
Impact of Stinging Jellyfish Proliferations along South Italian Coasts: Human Health Hazards, Treatment and Social costs	De Donno A, et al. 2014 [82]	Jellyfish (1733)		Ocular edema (22, 1%)			Urticaria (8, 0.5%)		Panic attacks (3, 0.2%) Anaphylactic shock (1, 0.1%)			(1, 0.1%)			Dyspnea (1, 0.1%)		Conjunctivitis (3, 0.2%)			Infection (1, 0.1%)

Title	Author, year published [ref.]	Organism(s)	Pain	Edema	Erythema	Fever	Pruritus	Malaise	Shock	Nausea and vomiting	Neuro symptoms	Muscular spasms	Non-specific systemic symptoms	Necrosis	Respiratory failure and pulmonary edema	Blurred vision/dizziness	Conjunctivitis	Wound soiling	Ulcers, lesions, or bleeding	Secondary infection
Marine envenomations in returning French travelers seen in a tropical diseases unit, 2008-13.	Henn A, et al. 2016 [83]	Corals (11)	(1, 9%)	(2, 18%)	(2, 18%)		(5, 45%)				Paresthesia (1, 9%)								Papular lesions (3, 27%) Vesicular lesions (3, 27%) Crust (1, 9%)	Superinfection (4, 36%)
		Stonefish (10)	(4, 40%)	(5, 50%)	(4, 40%)		(1, 10%)				Paresthesia (2, 20%)			(2, 20%)						Superinfection (9, 90%)
		Jellyfish (8)	(0, 0%)	(1, 13%)			(2, 25%)				Paresthesia (2, 25%)								Flagellation (6, 75%) Papular lesion (13%)	Superinfection (2, 25%)
		Miscellaneous (8)	(4, 50%)	(3, 38%)	(2, 18%)		(0, 0%)				Paresthesia (1, 13%)								Flagellation (1, 13%) Vesicular lesions (1, 13%) Crust (1, 13%)	Stingray (2, 100%), Weever fish (1, 50%), Starfish (2, 100%), Sea anemone (1, 100%) Lionfish (1, 100%)
**Africa**																				

Title	Author, year published [ref.]	Organism(s)	Pain	Edema	Erythema	Fever	Pruritus	Malaise	Shock	Nausea and vomiting	Neuro symptoms	Muscular spasms	Non-specific systemic symptoms	Necrosis	Respiratory failure and pulmonary edema	Blurred vision/dizziness	Conjunctivitis	Wound soiling	Ulcers, lesions, or bleeding	Secondary infection
Epidemiology of the cnidarian Pelagia noctiluca stings on Moroccan Mediterranean beaches	Mghili B, et al. 2020 [92]	Jellyfish	Yes		Yes		Yes													
Tropical marine faunal hazard knowledge, incidents and associated health burden among seascape users at the Kenyan coastline	Kihia CM, et al. 2023 [93]	Lionfish, stonefish, stingray, urchin, jellyfish sea snake	Yes (89%)		Yes (lionfish jellyfish, urchin)		Yes (59%)				Yes (stingray, stonefish, lionfish)								Ulcers (stingray, stonefish, lionfish)	Blackened skin (70%) Rotting (54%)
**Oceania**																				

Title	Author, year published [ref.]	Organism(s)	Pain	Edema	Erythema	Fever	Pruritus	Malaise	Shock	Nausea and vomiting	Neuro symptoms	Muscular spasms	Non-specific systemic symptoms	Necrosis	Respiratory failure and pulmonary edema	Blurred vision/dizziness	Conjunctivitis	Wound soiling	Ulcers, lesions, or bleeding	Secondary infection
Animal bite wounds and their management in tropical Australia	Vardanega J, et al. 2022 [97]	Jellyfish (129)																(1, 0.8%)		
		Stonefish (22)																(1, 5%)		Infected at presentation (4, 18%)<br>Deep infection (1, 5%)<br>Developed infection (1, 5%)
		Stingray (17)																(5, 29%)		Infected at presentation (8, 47%)<br>Deep infection (1, 13%)<br>Developed infection (0, 0%)

Title	Author, year published [ref.]	Organism(s)	Pain	Edema	Erythema	Fever	Pruritus	Malaise	Shock	Nausea and vomiting	Neuro symptoms	Muscular spasms	Non-specific systemic symptoms	Necrosis	Respiratory failure and pulmonary edema	Blurred vision/dizziness	Conjunctivitis	Wound soiling	Ulcers, lesions, or bleeding	Secondary infection
Australian Sea Snake Envenoming Causes Myotoxicity and Non-Specific Systemic Symptoms - Australian Snakebite Project (ASP-24)	Johnston C, et al 2022 [98]	Sea snake	Muscle pain (5, 38.5%)<br>Myotoxicity (2, 15.4%)<br>Abdominal pain (3, 23.1%)	(5, 38.5%)	Bruising (1, 7.7%)				Diaphoresis (2, 15.4%)	(6, 46.2%)	Headache (6, 46.2%)		(6, 46.2%)						Bleeding (2, 15.4%)	
